# How effective are anthocyanins on healthy modification of cardiometabolic risk factors: a systematic review and meta-analysis

**DOI:** 10.1186/s13098-023-01075-0

**Published:** 2023-05-23

**Authors:** Tirang R. Neyestani, Zahra Yari, Hamid Rasekhi, Bahareh Nikooyeh

**Affiliations:** Nutrition Research Department, National Nutrition and Food Technology Research Institute and Faculty of Nutrition Sciences and Food Technology, Shahid Beheshti University of Medical Sciences, Tehran, 198161957 Iran

**Keywords:** Anthocyanin, Meta-analysis, Cardiometabolic risk factors

## Abstract

**Background:**

In this meta-analysis, findings from recent studies on the preventive properties of anthocyanins (ACN) against cardiovascular disease are summarized.

**Methods:**

MEDLINE, PubMed, Embase, Cochrane Library, and Google Scholar were searched and 2512 studies were found in a preliminary search. After screening of titles and abstracts, 47 studies met the inclusion criteria (randomized clinical trial design and sufficient data of outcomes). Studies were excluded based on the following criteria: incomplete data; obscurely reported outcomes, or lack of control groups; and animal studies.

**Results:**

The results showed that intervention with ACNs resulted in a significant decrease in body mass index ((MD),− 0.21; 95% CI, − 0.38, − 0.04; P < 0.001) and body fat mass (MD: − 0.3%, 95% CI − 0.42 to − 0.18%, p < 0.001). Pooled data comparing ACN with control showed statistically significant effect on fasting blood sugar and HbA1c. However, the reductions were significantly more in the subjects with type 2 diabetes and in those who used ACN as supplement/extract. The subgroup analysis test showed that there was a significant effect of ACN on triglyceride concentrations, total cholesterol, LDL-C and HDL-C concentrations in all subgroups of participants (with vs. without dyslipidemia at baseline) and intervention type (supplement/extract vs. food). However, we did not observe any significant effect on apo A and apo B concentrations.

**Conclusions:**

ACN intake in the forms of natural foods and supplements can induce healthy changes in body fat mass, glycemic and lipidemic status and these effects are more prominent in the subjects with above-normal values. This meta-analysis was registered at http://www.crd.york.ac.uk/Prospero (Registration no. CRD42021286466).

**Supplementary Information:**

The online version contains supplementary material available at 10.1186/s13098-023-01075-0.

## Background

Cardiovascular diseases (CVD), comprising ischemic heart disease, stroke, heart failure, peripheral arterial disease, and a number of other cardiac and vascular conditions are still the leading contributors to disease burden worldwide. According to the World Health Organization (WHO) by 2025, over 28 million individuals will die from CVD annually [1, 2]. There is, therefore, an urgent need to develop and implement safe and cost-effective interventions and policies [3].

Over the past decade, there has been an increasing interest in lifestyle and dietary interventions to lessen chronic disease risks. A huge body of evidence shows that fruits and vegetables intake is inversely associated with the risk of the most common chronic diseases including CVD [4]. These effects are attributed to the abundance of bioactive compounds in fruits and vegetables [5].

Anthocyanins (ACN) seem to be the major subgroup of flavonoids (pigments occurring in fruits and vegetables) having exclusive metabolic and biological behaviors [6, 7]. There are more than 700 structurally distinct ACN derivatives that naturally occur in plants as anthocyanidin (aglycone) attached to sugar moieties [8]. Important sources of ACN in a typical American diet include berries, currants, grapes, wine and vegetable sources include (red) cabbages, radishes, onions, grains, roots and tubers [9].

A growing body of evidence indicates that consumption of ACN-rich products can reduce the risk of CVD, mainly in the subjects at risk [10, 11]. For example, a clinical research demonstrated that consumption of pomegranate juice, which contains ACN and tannins, lowers blood pressure and carotid intima media thickness [12]. Also, meta-analyses of prospective cohort studies suggest that high intakes of ACN may be associated with a decreased risk of CVD [13, 14] and type 2 diabetes (T2D) [15]. Though a meta-analytical study reported the beneficial impacts of ACN on cardiometabolic health some five years ago [16], current data are still conflicting [17, 18]. Furthermore, the possible effects of ACN on some important variables including waist circumference and body fat mass have not already been summarized and analyzed. In this meta-analysis, findings from recent studies on the preventive properties of ACN against CVD are summarized and current knowledge of the field is updated. To do this, we included 41 clinical trial studies and performed subgroup analysis and meta-regression for the cardiometabolic risk factors including body mass index, waist circumference and body fat mass, that have not been examined in the previous meta-analysis.

## Methods

This systematic review and meta-analysis was planned, conducted, and reported according to the widely accepted quality standards [19] and was registered at http://www.crd.york.ac.uk/Prospero (Registration no. CRD42021286466).

### Types of interventions and outcome measures

The interventions investigated in this review were giving ACN either as supplement or food in various doses and frequencies as compared with a control group. No limit was placed on the dose, type of vehicle or frequency at which supplements and/or fortified foods were taken.

Outcome measures were cardiometabolic risk factors including:

Anthropometric measures: weight (kg), body mass index (BMI) (kg/m^2^), waist circumference (WC) (cm), body fat mass (FM)(%).

Glycemic indicators: fasting blood glucose (FBG) (mg/dL), fasting serum insulin (FSI) (µIU/mL), two-hour glucose tolerance test (mg/dL), homeostasis model assessment-estimated insulin resistance (HOMA-IR), glycated hemoglobin (HbA1c) (%).

Lipid profile: serum triglyceride (TG) (mg/dL), total cholesterol (TC) (mg/dL), low density lipoprotein-cholesterol (LDL-C) (mg/dL), high density lipoprotein-cholesterol (HDL-C) (mg/dL), apolipoprotein A (Apo A) (mg/dL), apolipoprotein B (Apo B) (mg/dL).

### Search strategy

A comprehensive search of electronic databases was conducted for eligible trials up to September 2021. The following databases were covered: National Library of Medicine (PubMed), Scopus, Web of Science (WoS), and Cochrane Database of Systematic Reviews (Cochrane Library, CDSR) using the following search terms in titles and abstracts: 1- (anthocyanin) AND (Anthropometric measures: weight, body mass index (BMI), waist circumference (WC), fat mass (FM)) 2- (anthocyanin) AND (Glycemic indicators: fasting blood glucose (FBG), fasting serum insulin (FSI), two-hour glucose tolerance test, homeostasis model assessment-estimated insulin resistance (HOMA-IR), HbA1c), 3- (anthocyanin) AND (lipid profile: serum triglyceride (TG), total cholesterol (TC), low density lipoprotein-cholesterol (LDL-C), high density lipoprotein-cholesterol (HDL-C), apolipoprotein A (apo A), apolipoprotein B (apo B)).

All of the studies were limited to English language and those in humans. All the references in the papers of the included studies were scanned for further relevant reports.

### Types of studies: inclusion and exclusion criteria

Two investigators independently searched and assessed articles for eligibility according to the following inclusion criteria:

1. all studies with a randomized clinical trial design;
2. data description as mean ± SD and sufficient data of outcomes at both baseline and the end of intervention in each group or the net change values provided

Studies were excluded based on the following criteria:

1. incomplete data;
2. duplicate publication of articles;
3. obscurely reported outcomes, or lack of control groups; and
4. animal as well as non-interventional studies

### Data extraction and management

Two reviewing authors independently screened titles or abstracts to exclude studies that failed to meet the inclusion criteria and then obtained the full-text reports for further evaluations. Discrepancies were resolved through consensus. Detailed data of study design, context, participants’ information, interventions and outcomes were extracted. For multi-armed studies, pairs of arms relevant to the review were compared. Data of the control group were used for each intervention group comparison. The weight assigned to the control group was reduced by dividing the control group number (N) by the number of intervention groups. The selection process in sufficient detail to complete a PRISMA flow diagram was recorded in the supplementary data.

### Assessment of risk of bias in the included studies

In order to assess the risk of bias for each of the included studies, the two reviewing authors independently assessed the quality of the included studies according to the Cochrane risk of bias tool [20] and following criteria: (i) random sequence generation, (ii) allocation concealment, (iii) blinding of participants, (iv) blinding of personnel and outcome assessment, (v) incomplete outcome data, and (iv) selective outcome reporting and other bias (bias due to problems not covered elsewhere, e.g. industry funding).

### Assessment of reporting biases

Funnel plots were used to assess the potential existence of bias (were not shown). We performed a regression asymmetry test for detection of bias [21]. In this study, p < 0.05 was considered significant.

### Subgroup analysis and investigation of heterogeneity

If we identified substantial heterogeneity and there were sufficient data, we would investigate it using subgroup as well as sensitivity analyses. The following subgroup analyses were carried out:

- participants’ health status ( having diabetes, dyslipidemia, …)
- type of intervention (supplements/extract vs. food)

### Sensitivity analysis

The aim of the sensitivity analysis was to examine the effect size when some studies had less stringent inclusion criteria. If there were sufficient data to allow for sensitivity analysis, this analysis would be performed for examining the effects on results by excluding:

- (i) Trials at high risk of bias, as specified in the Assessment of risk of bias in included studies section.
- (ii) Trials with small sample sizes (less than 15 participants in each group);

Moreover, the leave-one-out analysis was performed to assess the key studies with substantial impact on between-study heterogeneity.

### Meta-regression

Random‐effects model meta‐regression analyses were performed to assess sources of inter-trial heterogeneity. The follow-up time, ACN dose and variables at baseline (according to model and dependent variable) were considered as covariates.

### Statistical analyses

Meta-analysis was performed using STATA version 17.0 (StataCorp, College Station, TX). For continuous outcomes, a mean difference (MD) and 95% confidence interval (CI) calculated for each study (i.e. intervention group minus control group differences).

The data reported as median were converted to mean and SD using the formula recommended by the Cochrane Collaboration Handbook [20].

In addition, heterogeneity was assessed using Q and I^2^ tests. The fixed effect model was used as pooling method when there was no statistically significant heterogeneity (I^2^ < 50%), whereas the random-effects model was employed on the contrary (I^2^ > 50%).

The Egger’s test was used to assess risk of publication bias.

## Results

Initially, 2,512 studies were found in a preliminary search from which 2,444 studies were excluded after removing duplicates and primary screening of titles and abstracts, another 21 studies were excluded due to following reasons:

- 1) There was no control group;
- 2) The studies provided no available data.

**Fig. 1:**
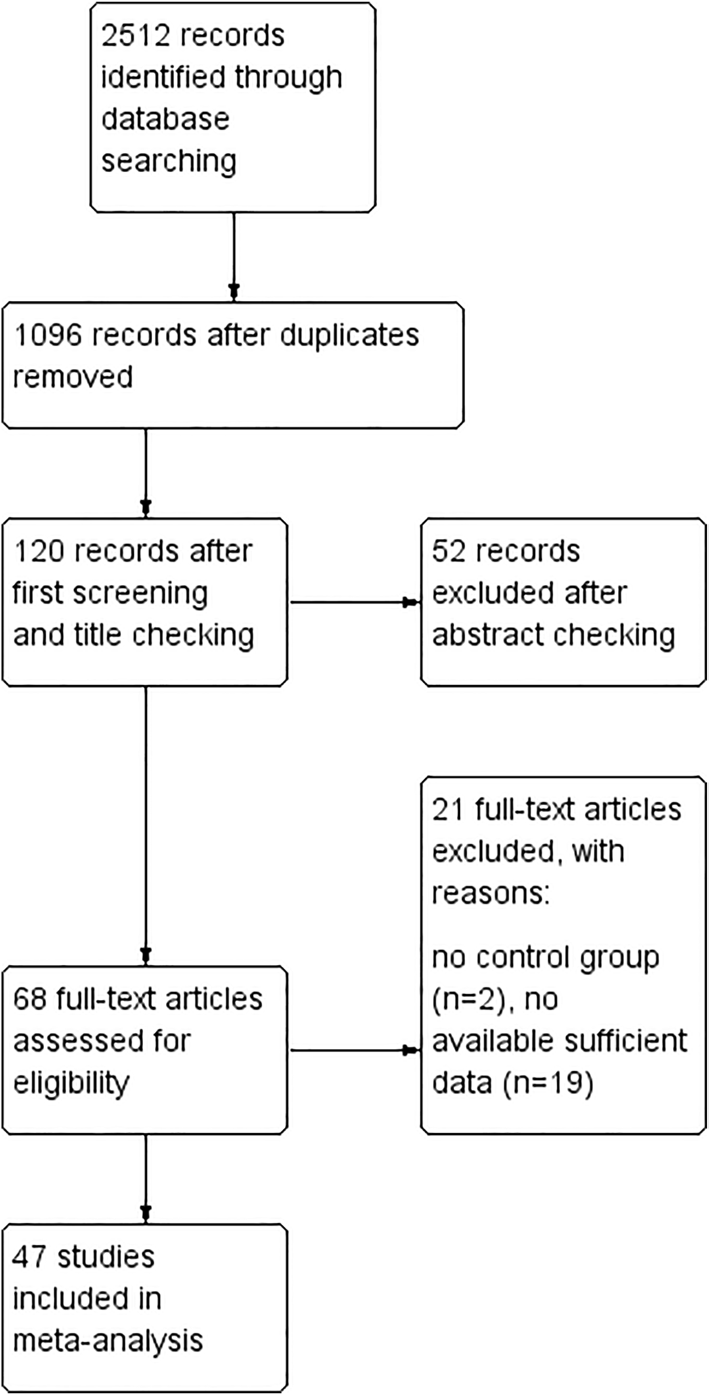
The flowchart of the study protocol on the effects of anthocyanins intake on cardiometabolic risk factors

Finally, a total of 47 studies were selected for the analysis. A flow diagram showing the selection process is presented in Fig. 1.

### Characteristics and bias risk assessment of included studies

The basic characteristics of the 47 randomized clinical trials (RCTs) are listed in Table 1. There were eight studies in healthy participants [22–29], four in prediabetic adults [30–33], eight in subjects with metabolic syndrome [34–41], one in elderly with mild to moderate dementia Alzheimer [42], four in overweight/obese subjects [43–46], one in adults with cardiovascular risk factors [47], seven in patients with T2D [48–54], eight in dyslipidemia [55–62], one in subjects with non-alcoholic fatty liver disease [63] and three in patients with ischemic coronary heart disease [64–66]. Trial intervention duration ranged from 3 weeks [46] to 24 months [50]. The sample size from all the trials ranged between 20 [29, 54, 67] and 160 participants [32, 33].

**Table 1:** Characteristics of the studies selected for meta-analysis of the effects of anthocyanins on cardiometabolic risk factors

Study	Country year	Participants	Type and dose of anthocyanin	Duration (week)	Sample size
Alnajjar M, et al. [46]	UK 2020	Overweight male and postmenopausal female, BMI 31 ± 4, aged 62 ± 7, with or without diabetes, were not on any special religious or prescribed diet, with stable weight, without thromboembolic or coagulation problems, thyroid disease, renal or hepatic disease, severe gastrointestinal disorders, pulmonary disease (e.g., chronic bronchitis, COPD), alcohol or any other substance abuse, eating disorders or psychiatric disorders, not were taking oral steroids, tricyclic antidepressants, neuroleptics anticoagulants, digoxin and anti-arrhythmic, or chronically using anti-inflammatory (e.g., high doses of aspirin, ibuprofen)	Three capsules of 0.47 g of Mirtoselect® [a standardized bilberry extract (36% (w/w) anthocyanins)] per day, which equates to ~ 150 g of fresh bilberries per day, formulated in gelatin capsules	3	Total: 16, with (n = 9) or without (n = 7) type 2 diabetes two groups: Control group: 16 Anthocyanin group: 16 Lost to follow-up: 0
An JH, et al. [30]	Korea 2016	Aged 20–80 years with prediabetes with no history of renal impairment, liver disease, uncontrolled hypertension, triglyceride > 500 mg/dL, other acute or chronic disease, no starting or changing lipid-lowering agent within two months, no take of medications known to affect glycemic control and lipid profiles or herbal supplements	Rubus occidentalis extract (RO, Black raspberry) Definite anthocyanins levels of RO extract investigated in this study were not available	12	Total: 44, three groups: Control group: 1 900 mg RO extract: 1 1800 mg RO extract: 17 Lost to follow-up: 5
Asgary S, et al. [68]	Iran 2014	hypertensive patients aged 30–67 years with BMI ≤ 30, without type 1 or 2 diabetes, chronic pancreatitis, liver cirrhosis, kidney stones, renal failure, use of non-steroidal anti-inflammatory drugs, use of antioxidant or vitamin supplements, intense physical activity (> 5 h/week), smoking habit, being vegetarian or having any restrictive dietary requirements and pregnancy	Pomegranate juice (PJ) Definite anthocyanins levels of PJ investigated in this study were not available	2	Total: 21, two groups: Control group: 10 Pomegranate juice group: 11 Lost to follow-up: 0
Bakuradze T, et al. [24]	Germany 2019	Healthy male volunteers, aged 20–50, BMI = 19–25, non-smokers, no practice of excessive sports, no intake of pharmaceutical drugs or food supplements	750 mL bolus of anthocyanin-rich fruit juice Total anthocyanins: 274.5 mg/L	8	Total: 62, two groups: Control group: 31 fruit juice group: 31 Lost to follow-up: 5
Basu A, et al. [34]	USA 2010	Obese adults with metabolic syndrome, older than 21 years, not using hypoglycemic, hypo lipidemic, anti-inflammatory, or steroidal medications; no history of liver, renal, or thyroid disorders or anemia, not consuming antioxidants or fish oil supplements on a regular basis	50 g freeze-dried blueberries that were reconstituted in 480 mL water and vanilla extract Total anthocyanins: 742 mg	8	Total: 66, two groups: Control group: 32 blueberry group: 34 Lost to follow-up: 18
Basu A, et al. [35]	USA 2010	Adults with metabolic syndrome, no medications for any chronic disease (cancer, congenital heart disease, diabetes mellitus), or medications known to affect lipid metabolism or possess anti-inflammatory effects, pregnant or lactating, used any form of tobacco products, consumed alcohol on a regular basis, used mega doses of antioxidants or fish oil supplements (> 1 g/day), or had any abnormalities in hematology, liver, renal, and thyroid function tests	Strawberry beverage Total anthocyanins: 154 mg	8	Total: 30, two groups: Control group: 15 Strawberry beverage group: 15 Lost to follow-up: 3
Basu A, et al. [36]	USA 2011	Adults with metabolic syndrome, no medications for any chronic disease (cancer, cardiovascular disease, diabetes mellitus), pregnant or lactating, used any form of tobacco products, consumed alcohol (> 1 oz/day), used mega doses of antioxidants or fish oil supplements (> 1 g/day), or had any abnormalities in hematology, liver, renal, and thyroid function tests	480 mL cranberry juice Total anthocyanins: 12.4 mg	8	Total: 36, two groups: Control group: 18 cranberry juice group: 18 Lost to follow-up: 5
Basu A, et al. [37]	USA 2014	Hyperlipidemic adults with abdominal adiposity and fasting serum lipids that met any 2 of the following 4 criteria: (1) total cholesterol > 200 mg/dL; (2) LDL-C > 100 mg/dL; (3) TGs > 150 mg/dL; and (4) HDL-C < 50 mg/dL for women and < 40 mg/dL for men Without medications for any chronic disease (cancer, cardiovascular disease, diabetes mellitus), pregnant or lactating, used any form of tobacco products, consumed alcohol (> 1 oz/day), used mega doses of antioxidants or fish oil supplements (> 1 g/day), no abnormalities in hematology, liver, renal, and thyroid function tests	474 mL of freeze-dried strawberries (FDS) beverages Total anthocyanins: low-dose FDS: 78 mg high-dose FDS: 155 mg	12	Total: 66, four groups: low-dose control: 15 low-dose FDS: 15 high-dose FDS: 15 high-dose control: 15 Lost to follow-up: 6
Chan SW, et al. [54]	China 2021	Patients with type 2 diabetes, with HbA1c ≥ 7%, controlled with oral hypoglycemic agents, BMI > 23 kg/m^2^, with no evidence of cardiovascular disease, overt vascular complications, evidence of renal, liver disease, not taking other nutritional supplements	Spray-dried water/ethanol extract of European Bilberry (Vaccinium vitis-idaea L.) containing ≥ 25% anthocyanidins 4 capsules – 350 mg Total anthocyanins: 1.4 g	4	Total: 20, two groups: Control group: 20 Anthocyanin group: 20 Lost to follow-up: 0
Kent K, et al. [42]	Australia 2017	Elderly with mild to moderate dementia Alzheimer without uncontrolled hypertension, uncontrolled diabetes, any other unstable physical or mental health condition or dysphagia	200 mL anthocyanin-rich cherry juice Total anthocyanins: 138 mg	12	Total: 49, two groups: Control group: 25 cherry juice group: 24 Lost to follow-up: 7
Curtis PJ, et al. [23]	United kingdom 2009	Healthy postmenopausal women, not taking hormone replacement therapy for ≥ 6 mo, BMI: 20–32, nonsmokers, No history of diabetes; hepatic, renal, cardiac, pulmonary, digestive, hematological, neurological, thyroidal, or psychiatric disease; or taking anti-inflammatory or steroidal medication, or vaccines and antibiotics	Elderberry extract capsule Total anthocyanins: 500 mg	12	Total: 57, two groups: Control group: 29 anthocyanin group: 28 Lost to follow-up: 5
Davinelli S, et al. [43]	Italy 2015	Overweight smokers without history of chronic diseases, no taking anti-inflammatory drugs, cardiovascular medications, lipid-altering drugs, and hormone replacement therapy	3 capsules of 150 mg standardized maqui berry extract Total anthocyanins: 162 mg	6	Total: 42, two groups: Control group: 16 Anthocyanin group: 26 Lost to follow-up: 0
Dohadwala, et al. [66]	USA, 2011	Patients with stable coronary artery disease, confirmed by angiography, exercise test, or documented history of myocardial infarction, without body weight > 115 kg, congestive heart failure, or acute medical illness, not taking vitamins exceeding 2 times the Recommended Dietary Allowance or food supplements within 1 mo of study	Cranberry juice (480 mL/d) Total anthocyanins: 94 mg	4	Total: 47, two groups: Control group: 23 Anthocyanin group: 24 Lost to follow-up: 3
Duthie SJ, et al. [67]	United kingdom 2006	healthy female, without taking medication or vitamin and mineral supplements	750 ml of cranberry juice Total anthocyanins: 2.8 mg/l	2	Total: 20, two groups: Control group: 9 Anthocyanin group: 11 Lost to follow-up: 0
Erlund I, et al. [47]	Finland 2008	Adults with cardiovascular risk factors, at least one of the following conditions: mild hypertension, elevated blood glucose, elevated serum total cholesterol or triacylglycerol, and low HDL cholesterol Without regular use of medications (except hormone replacement therapy) or dietary supplements, smoking, intestinal disorders, obesity, and vegetarianism	Whole bilberries (100 g) and a nectar containing 50 g crushed lingonberries, Black currant or strawberry purée (100 g, containing 80% black currants) and cold-pressed chokeberry and raspberry juice (0.7 dL juice, containing 80% chokeberry) Total anthocyanins: 515 mg	8	Total: 72, two groups: Control group: 36 Berry group: 36 Lost to follow-up: 0 (one excluded from analysis)
Gurrola-Díaz CM, et al. [65]	Mexico 2010	Individuals with and without metabolic syndrome, aged 30–71 years	100 mg Hibiscus sabdariffa extract powder (HSEP) Total anthocyanins: 19.24 mg	4	Total: 152, six groups: Healthy Control (without metabolic syndrome): Diet group:27 HSEP group:26 Diet + HSEP group: 20 Lost to follow-up: 7 Metabolic syndrome group: Diet group:11 HSEP group:18 Diet + HSEP group: 22 Lost to follow-up: 21
Jeong HS, et al. [38]	Korea 2014	Patients with metabolic syndrome, 18 and 75 years old	750 mg Black Raspberry Definite anthocyanins levels of RO extract investigated in this study were not available	12	Total: 77, two groups: Control group: 38 Black raspberry group: 39 Lost to follow-up: 4
Kianbakht S, et al. [48]	Iran 2013	diabetic outpatients aged 40–60 years, resistant to conventional oral anti-hyperglycemic drugs, with FBG: 200–250 mg/dl and HbA1c: 7- 8, despite using a combination of conventional oral anti-hyperglycemic drugs, without cardiac, renal, or hepatic diseases or concomitant infections	350 mg whortleberry fruit hydroalcoholic extract Total anthocyanins: 86.56 mg / 10 g extract	8	Total: 86, two groups: Control group: 44 Whortleberry group: 42 Lost to follow-up: 12
Kianbakht S, et al. [55]	Iran 2014	Outpatients aged 20–60 years with newly diagnosed primary hyperlipidemia without taking anti-hyperlipidemic agents; herbal medications; triglyceride or/and total cholesterol > 350 mg/dL; no history of cardiac, renal, hepatic and hematological diseases, hypothyroidism, diabetes mellitus, tachycardia, vertigo and seizure; gallstones or gall bladder surgery; not using estrogen, steroid, betablocker and thiazide;	350 mg whortleberry fruit hydroalcoholic extract Total anthocyanins: 86.56 mg / 10 g extract	8	Total: 105, two groups: Control group: 54 Whortleberry group: 51 Lost to follow-up: 25
Kim H, et al. [39]	USA 2018	metabolic syndrome patients, without previous hospitalizations due to metabolic syndrome, current pregnancy or lactation, recent history of significant weight change, weight loss medications, dietary supplements, smoking, binge drinking, and allergy to açaí berries	325 mL açaí beverage Total anthocyanins: 307 mg/L	12	Total: 43, two groups: Control group: 22 açaí group: 21 Lost to follow-up: 6
Lee I, et al. [49]	Taiwan 2018	Type 2 diabetic subjects, 50–75 years old, HbA1c 7–10%, triglycerides < 4.5 mmol/l Without kidney or hepatic disease, severe systemic disease such as immune disorders, cancer, acute or chronic inflammatory disease; smoking, insulin or steroid use	500 mg cranberry extract Definite anthocyanins levels of RO extract investigated in this study were not available	12	Total: 30, two groups: Control group: 15 Cranberry group: 15 Lost to follow-up: 0
Lee M, et al. [44]	Korea 2016	19–65 years old with BMI > 23, and waist circumference > 90 cm (male) or 85 cm (female). Without using anti-obesity or lipid-lowering medication, no history of cardiovascular or obesity-related diseases, no more than 10% loss of body weight within the previous 3 months	2.5 g Black soybean testa extracts (BBT) Total anthocyanins: 32 mg	8	Total: 80, two groups: Control group: 31 BBT group: 32 Lost to follow-up: 17
Li D, et al. [50]	China 2015	Type 2 diabetes aged 56–67 years, without recently diagnosed diabetes, pregnancy, coronary artery disease, and other known chronic diseases	Anthocyanin capsules Total anthocyanins: 320 mg	24	Total: 58, two groups: Control group: 29 Anthocyanin group: 29 Lost to follow-up: 0
Lynn A, et al. [22]	United Kingdom 2014	Healthy, non-smoking, age 30–50 y, without taking medication for heart disease, hypertension or diabetes, regular consumption of cherries or cherry juice and the use of antioxidant supplements	Cherry juice concentrate [30 ml diluted with 220 ml of water] Total anthocyanins: 273.5 mg	6	Total: 47, two groups: Control group: 21 Cherry juice group: 25 Lost to follow-up: 1
Murkovic M, et al. [25]	Austria 2004	Healthy volunteers not regularly taking antioxidant supplements	Spray-dried elderberry juice capsule Total anthocyanins: 100 mg	2	Total: 34, two groups: Control group: 17 Cherry juice group: 17 Lost to follow-up: 0
Naruszewicz M, et al. [56]	Poland 2017	Patients who survived myocardial infraction and have received statin therapy for at least 6 months	3 × 85 mg/day of chokeberry flavonoid extract Total anthocyanins: 25%	6	Total: 44, two groups: Control group: 22 Chokeberry group: 22 Lost to follow-up: 0
Novotny JA, et al. [26]	USA 2015	nondiabetic, nonsmokers with fasting TGs < 300 mg/dL not using cholesterol-lowering medications, BP medications, history of bariatric or weight-loss surgery, recent weight loss, history of gastrointestinal or malabsorption disorders, kidney disease, liver disease, gout, hyperthyroidism, untreated or unstable hypothyroidism, cancer, pancreatic disease, or other metabolic disorders	240 mL of low-calorie cranberry juice Total anthocyanins: 10.3 mg	8	Total: 60, two groups: Control group: 30 Cranberry group: 30 Lost to follow-up: 4
Puupponen‐Pimiä R, et al. [40]	Finland 2013	Metabolic syndrome	300 g fresh berries comprising of 100 g of strawberry puree, 100 g of frozen raspberries, and 100 g of frozen cloudberries Total anthocyanins: 70.7 ± 52 mg	8	Total: 37, two groups: Control group: 17 Berry group: 20 Lost to follow-up: 5
Qin Y, et al. [57]	China 2009	Dyslipidemiac subjects met 2 of the following 4 criteria: TC > 200 mg/dL, TGs > 150 mg/dL, LDL-C > 100 mg/dL, HDL-C < 40 mg/dL with no history of CVD or other severe chronic disease or use of any drugs known to affect lipid metabolism	Anthocyanins capsule Total anthocyanins: 320 mg	12	Total: 120, two groups: Control group: 60 Anthocyanins group: 60 Lost to follow-up: 5
Riso P, et al. [29]	Italy, 2013	Healthy male subjects, aged 47.8 ± 9.7 years with BMI 24.8 ± 2.6 kg/m^2^, with at least one risk factor for CVD based on American Heart Association guidelines without secondary hypertension, obesity, diabetes, renal insufficiency, known food allergies, chronic constipation, diarrhea or any other gastrointestinal problem or disease, not taking drugs, dietary supplements, specific probiotics, prebiotics or medications, not taking high ([5 portions/day) or low (\2 portions/day) intake of fruit and vegetables and habitual alcohol consumption (\3drinks per week), not following a specific diet	Wild blueberry (WB) drink was prepared by suspending 25 g of WB freeze-dried powder in 250 mL of water Total anthocyanins: 375 mg	6	Total: 20, two groups: Control group: 20 Anthocyanin group: 20 Lost to follow-up: 2
Shidfar F, et al. [51]	Iran 2012	Nonsmoking T2D men with BMI < 30 kg/m, HbAlc < 9%, serum triglyceride < 400 mg/dl, and serum total cholesterol < 240 mg/dl, with no history of symptomatic heart disease, myocardial infarction, angina pectoris or stroke, surgery, liver, renal, or thyroid disease; use of steroids or change in the use of medication	240 ml cranberry juice Definite anthocyanins levels of RO extract investigated in this study were not available	12	Total: 60, two groups: Control group 30 Cranberry group: 30 Lost to follow-up: 2
Soltani R, et al. [58]	Iran 2014	hyperlipidemic patients with TC: 200–300 mg/dL and/or TGs: 150–199 mg/dL and/or LDL-C: 130–190 mg/dL, being non-smokers, free of diseases affecting serum lipids (e.g., diabetes, thyroid disorders, and pancreatitis), not using drugs or supplements affecting serum lipids (e.g., statins, fibrate derivatives, estrogens, progestins, beta-blockers, thiazide diuretics, and fish oil) within the last 3 months, free of liver or kidney disease,	Fruit extract of Vaccinium arctostaphylos L. capsule Total anthocyanins: 90 mg	4	Total: 54, two groups: Control group: 27 Vaccinium group: 27 Lost to follow-up: 4
Soltani R, et al. [52]	Iran 2015	T2D patients, being diagnosed for at least 2 years, age of 18–80 years, HbA1c 7–10%, not being alcohol or substance abuser, not using insulin or insulin secretagogue	Anthocyanins capsule Total anthocyanins: 600 mg	6	Total: 60, two groups: Control group: 30 Anthocyanin group: 30 Lost to follow-up: 0
Stote KS, et al. [53]	USA 2020	Men diagnosed with T2D for ≥ 6 mo; HbA1c: 6.5–9; BMI > 25. Without chronic kidney disease, liver cirrhosis, gastrointestinal disease, pancreatic disease, or malabsorption syndromes, losing > 10% of weight within the past 12 mo, participating in a heavy exercise program	22 g freeze-dried blueberries Total anthocyanins: 261.8 mg	8	Total: 55, two groups: Control group: 28 Blueberry group: 27 Lost to follow-up: 3
Stull AJ, et al. [45]	USA 2010	Obese, nondiabetic, and insulin-resistant subjects, BMI: 32–45 kg/m^2^, without history of diabetes, thyroid or chronic liver, renal, or cardiovascular disease, allergic reactions to blueberries, not using medications known to affect glucose metabolism; drug and/or alcohol abuse, not consuming berries, grapes, and wine > 3 times/wk; and > 5% fluctuation in body weight preceding 2 mo	Smoothie containing 22.5 g blueberry bioactives with 45 g of blueberry powder Total anthocyanins: 668 mg	6	Total: 32, two groups: Control group: 17 Blueberry group: 15 Lost to follow-up: 0
Stull AJ, et al. [41]	USA 2015	Metabolic syndrome patients without diabetes, liver, kidney or heart disease, smoking, not using nonprescription drugs, vitamins, dietary and herbal supplements	Skim milk-based smoothie with 45 g freeze-dried blueberry powder added Total anthocyanins: 290.3 mg	6	Total: 46, two groups: Control group: 23 Blueberry group: 23 Lost to follow-up: 2
Sumner MD, et al. [64]		Patients with ischemic coronary heart disease with ≥ 1 reversible myocardial perfusion defect without history of other heart diseases or significant co-morbidity, current use of tobacco products, or alcohol or drug abuse	240 mL of pomegranate juice Definite anthocyanins levels of RO extract investigated in this study were not available	12	Total: 45, two groups: Control group: 26 Pomegranate group: 19 Lost to follow-up: 4
Wright OR, et al. [27]	Australia 2013	Healthy male volunteers aged 18–65 years with a BMI 25–35 with stable blood pressure in the normal, high-normal, or mildly hypertensive or being stable on medication if are hypertensive; with no chronic diseases or medical conditions, not already consuming purple carrots or purple carrot products	Sachets of dried purple carrot Total anthocyanins: 118.5 mg	4	Total: 16, two groups: Control group: 8 Purple carrot group: 8 Lost to follow-up: 0
Xu Z, et al. [59]	China 2020	Dyslipidemic subjects	Purified anthocyanin capsules Total anthocyanins: 40–80-320 mg	12	Total: 176, four groups: Control group: 46 Anthocyanin (40 mg):45 Anthocyanin (80 mg):42 Anthocyanin (320 mg):43 Lost to follow-up: 5
Yang L, et al. [32]	China, 2020	Prediabetes or early untreated diabetes, aged 40–75 years, HbA1c: 5.7–6.4%, without medical history of diabetes, untreated thyroid disease, polycystic ovarian syndrome, serious liver or kidney dysfunction, infection, or traumatic injury or surgeries, not using glucocorticoids	Purified anthocyanins Total anthocyanins: 230 mg	12	Total: 160, two groups: Control group: 80 Anthocyanin group: 80 Lost to follow-up: 22
Yang L, et al. [33]	China, 2021	Prediabetes or early untreated diabetes, aged 40–75 years, HbA1c: 5.7–6.4%, without medical history of diabetes, untreated thyroid disease, polycystic ovarian syndrome, serious liver or kidney dysfunction, infection, or traumatic injury or surgeries, not using glucocorticoids	Purified anthocyanins Total anthocyanins: 230 mg	12	Total: 160, two groups: Control group: 80 Anthocyanin group: 80 Lost to follow-up: 22
Yang L, et al. [31]	China 2017	Prediabetes or early untreated diabetes, aged 40–75 years, HbA1c: 5.7–6.4%, without medical history of diabetes, untreated thyroid disease, polycystic ovarian syndrome, serious liver or kidney dysfunction, infection, or traumatic injury or surgeries, not using glucocorticoids	Purified anthocyanins Total anthocyanins: 230 mg	12	Total: 160, two groups: Control group: 80 Anthocyanin group: 80 Lost to follow-up: 22
Yarahmadi M, et al. [28]	Iran 2014	athletes with athletic history of at least 3 years, not using antioxidant supplements during the past month	Anthocyanins pill Total anthocyanins: 100 mg	6	Total: 54, two groups: Control group: 27 Anthocyanin group: 27 Lost to follow-up: 10
Zhang P-W, et al. [63]	China 2015	NAFLD patients, 25–65 years, without excessive alcohol consumption, cirrhosis, viral hepatitis, cardiovascular disease, cancer, and any consumption of nonsteroidal anti-inflammatory drugs, corticosteroids, or prescriptive medicines that affect liver function, lipid, or glucose metabolism	Purified anthocyanins from bilberry and black currant Total anthocyanins: 320 mg	12	Total 74, two groups: Control group: 37 Anthocyanin group: 37 Lost to follow-up: 11
Zhang X, et al. [60]	China 2016	Hypercholesterolemic individuals, with total cholesterol:200–310 mg/dL, without a history of CVD, hypertension, diabetes mellitus, thyroid disorder, smoking or the use of any drug that could influence the measurement of lipid parameters, inflammatory markers, or chemokines	Purified anthocyanins Total anthocyanins: 320 mg	24	Total: 150, two groups: Control group: 75 Anthocyanin group: 75 Lost to follow-up: 4
Zhao Y, et al. [61]	China 2021	Dyslipidemic subjects, 35–70 years, without history of atherosclerosis, coronary disease, or other severe vascular disease, infection, autoimmune disease, cancer, traumatic injury, or surgery within 1 month; liver or renal dysfunction; prescription of lipid-lowering medications within 6 months; intake of phytochemical supplements including anthocyanin and grape seed extracts within 2 months	Purified anthocyanin concentrated from skins of wild Norwegian bilberries and black currants Total anthocyanins: 40–80-320 mg	12	Total: 176, four groups: Control group: 46 Anthocyanin (40 mg):45 Anthocyanin (80 mg):42 Anthocyanin (320 mg):43 Lost to follow-up: 7
Zhu Y, et al. [62]	China 2013	Hypercholesterolemic adults, aged 40–65 y, with total cholesterol:200–310 mg/dL, without history of cardiovascular disease, diabetes, hypertension, thyroid disorders, smoking or the use of any drugs that could influence lipid parameters and inflammatory markers	Purified anthocyanin mixture Total anthocyanins: 320 mg	24	Total: 150, two groups: Control group: 75 Anthocyanin group: 75 Lost to follow-up: 4

*BBT* black soybean testa; *BMI* body mass index; *BP* blood pressure; *CVD* cardiovascular disease; *FBG* fasting blood glucose; *FDS* freeze-dried strawberries; *HbA1c* glycated hemoglobin; *HDL-C* high density lipoprotein-cholesterol; *HSEP* Hibiscus sabdariffa extract powder; *LDL-C* low density lipoprotein-cholesterol; *NAFLD* non-alcoholic fatty liver disease; *PJ* pomegranate juice; *RO* Rubus occidentalis extract; *TC* total cholesterol; *T2D* type 2 diabetes; *TGs* triglycerides

A total of 23 trials compared provision of ACN supplement/extract in comparison with placebo or no intervention [23, 25, 28, 31–33, 43, 44, 46, 48–52, 54–63, 65].

#### Risk of bias in included studies

All trials provided data on losses to follow-up; eleven studies reported losses of > 10% [28, 30, 31, 34, 36, 40, 42, 44, 48, 55, 65] (Additional file 1: Figures S1 and S2). In thirteen trials, the information on the methods used for allocation concealment was unclear [23, 24, 30, 34–38, 42, 43, 47, 67, 68]. Three trials reported small sample size (< 15 subjects in each arm) [27, 67, 68].

#### Effect of anthocyanin on body mass index

Changes of BMI (kg/m^2^) after intervention with ACN were evaluated in twenty studies. From two studies that had multi-arm intervention, two pairs of arms were included in the analysis [30, 37]. Four studies included participants who were overweight or obese at baseline [27, 43–45].

The results of twenty trials showed that intervention with ACN resulted in a significant decrease in BMI (1,280 participants, mean difference (MD),− 0.21; 95% CI, − 0.38, − 0.04; P < 0.001) (Additional file 1: Figure S3).

The meta-regression analysis (random effects and maximum likelihood) with the difference of means as the dependent variable and the follow-up time (β = 0.01; 95% CI − 0.02, 0.042; p = 0.484), ACN dose (β = 0.00; 95% CI − 0.001, 0.00; p = 0.466) and BMI at baseline (β = 0.23; 95% CI − 0.04, 0.09; p = 0.490) as covariates showed no significant effects.

**Fig. 2:**
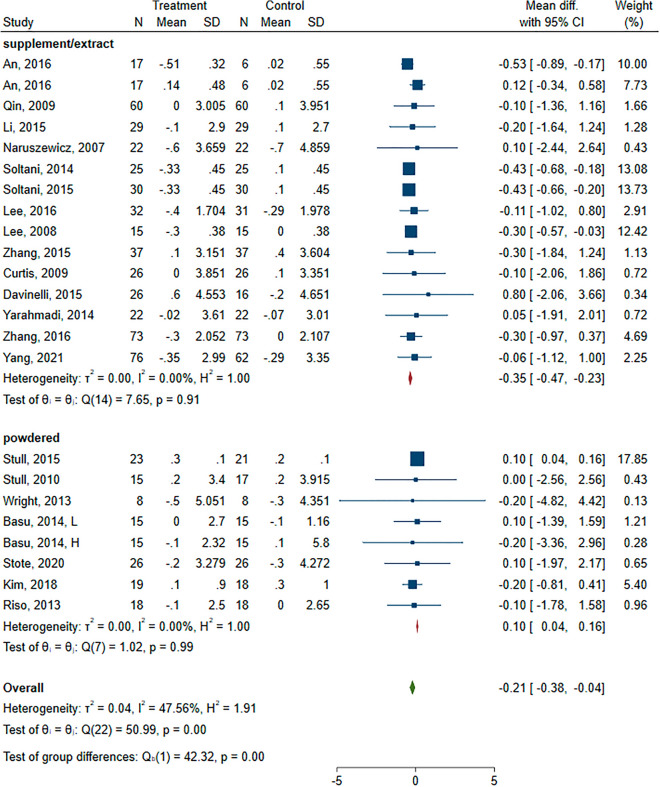
Forest plot of subgroup analysis of the effect of anthocyanins compared with control on body mass index (kg/m^2^)

After conducting stratified analyses according to the type of ACN (supplement/extract vs. food), we found a statistically significant effect in both subgroups (Fig. 2). However, unlike supplement (MD: − 0.35; 95% CI − 0.47, − 0.23; P < 0.001), using ACN as powdered or food was accompanied by an increase in BMI in the intervention group as compared with control (MD: 0.1, 95% CI 0.04, 0.16; P = 0.001).

Sensitivity analyses were performed and made no remarkable change in overall results (MD: − 0.20, 95% CI − 0.39, − 0.04, P = 0.01). The results of leave-one-out analysis were shown in the (Additional file 1: Figure S4).

The Egger’s tests indicated no significant publication bias (P = 0.605).

#### Effect of anthocyanin on waist circumference

Eight trials compared the effect of ACN intake, as supplement/foods, versus control on waist circumference (WC). From one trial, two paired-arms were included in the analysis. Pooled data showed no statistically significant effect on WC (614 participants, MD: 0.78 cm, 95% CI − 0.43, 1.98 cm; P = 0.21) (Additional file 1: Figure S5).

Sensitivity analysis was performed for examining the effects of excluding trials with small sample sizes. The results were shown no significant effect (MD: − 0.09 cm, 95% CI − 1.25, 1.07; P = 0.88).

The results of leave-one-out analysis were shown in the (Additional file 1: Figure S6).

### Effect of anthocyanin on body fat mass

Four trials compared the effect of ACN intervention with control on body fat mass (FM). Pooled data showed statistically significant effect on FM changes (150 participants, MD: − 0.3%, 95% CI − 0.42 to − 0.18%, p < 0.001) (Additional file 1: Figure S7).

The meta-regression analysis (random effects and maximum likelihood) with the difference of means as the dependent variable and the follow-up time (β = 0.01; 95% CI − 0.24, 0.27, p = 0.916) and used dose of ACN (β = 0.001; 95% CI − 0.01, 0.014, p = 0.853) as covariates showed the insignificant effects.

There were no data to allow for sensitivity analysis. The Egger’s tests indicated no significant publication bias (P = 0.881).

The results of leave-one-out analysis were shown in the (Additional file 1: Figure S8).

#### Effect of anthocyanin on fasting blood glucose

We pooled and analyzed data from 28 RCTs evaluating the effects of ACN intake on FBG. From three studies that had multi-arm intervention, two pairs of arms were included in the analysis [30, 37]. The forest plots for the effects of ACN intake on FBG are shown in the Additional file 1: Figure S9. The pooled MD was − 2.55 mg/dL (95%CI − 4.23, − 0.87), indicating a significantly greater reduction in FBG concentrations in the ACN intervention group than in the control (placebo) group.

The subgroup analyses were carried out to explore the impact of type of intervention (supplement/extract vs. food) and health status of participants (with diabetes vs. without diabetes). Significant reductions were observed in both subgroups of T2D patients (434 participants, MD: − 15.33 mg/dL; 95% CI − 25.8, − 7.86 mg/dL; P < 0.001) and participants without diabetes (1124 participants, MD: − 1.55 mg/dL; 95% CI − 2.38, − 0.72 mg/dL, P = 0.001). However, reduction of serum glucose concentrations was significantly more in the subjects with T2D (p < 0.001).

**Fig. 3:**
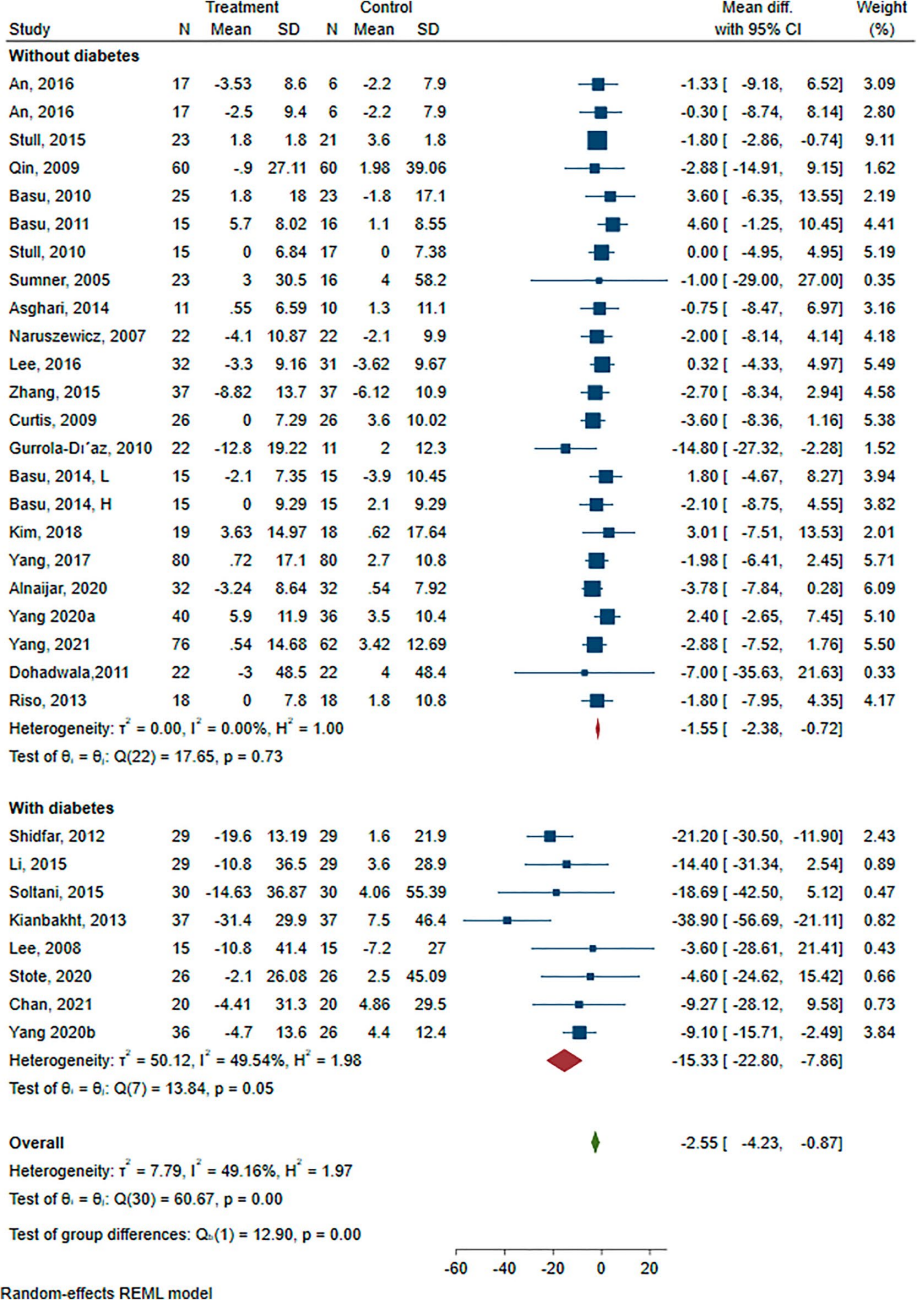
Forest plot of subgroup analysis of the effect of anthocyanins compared with control on fasting blood glucose (mg/dL)

After a subgroup analysis, we found that FBG decreased significantly only in those who used ACN as supplement/extract (MD: − 3.13 mg/dL, 95% CI − 4.9, − 1.35 mg/dL; P = 0.004) (Fig. 3).

The univariate meta-regression suggested that a higher baseline FBG was associated with a better effect of ACN intake on FBG changes (β = − 0.25; 95% CI − 0.34, − 0.15; P < 0.001) and this association remained significant after adjusting for duration of intervention, age of participants, dose of ACN and baseline BMI (β = − 0.25; 95% CI − 0.37, − 0.12; P < 0.001).

Sensitivity analyses were performed to test the robustness of the results and made no remarkable change in overall results (MD: − 3.0 mg/dL, 95%CI − 5.6, − 0.4 mg/dL, P = 0.02).

The results of leave-one-out analysis were shown in the (Additional file 1: Figure S10).

The Egger’s tests suggested that no significant publication bias (P = 0.089).

#### Effect of anthocyanin on fasting serum insulin

Eleven randomized controlled trials (two paired-arms from two studies) (n = 653) had measured fasting insulin. Results showed no significant effect of ACN intake on fasting insulin (MD: 0.64 µIU/mL; 95% CI − 0.33, 1.61 µIU/mL; P = 0.19) (Additional file 1: Figure S11).

A subgroup analysis was conducted to investigate heterogeneity using known factors that might influence circulating insulin, including participants (with vs. without diabetes) and intervention type (supplement/extract vs. food). Analyses did not show any significant influence (Additional file 1: Figure S12).

Results of sensitivity analysis showed no significant effect of ACN intake on fasting insulin (MD: 0.064 µIU/mL; 95% CI − 92, 1.09 µIU/mL; P = 0.87). The regression asymmetry test was not significant (p = 0.413).

The results of leave-one-out analysis were shown in the supplementary file (Additional file 1: Figure S13).

#### Effect of anthocyanin on HbA1c

**Fig. 4:**
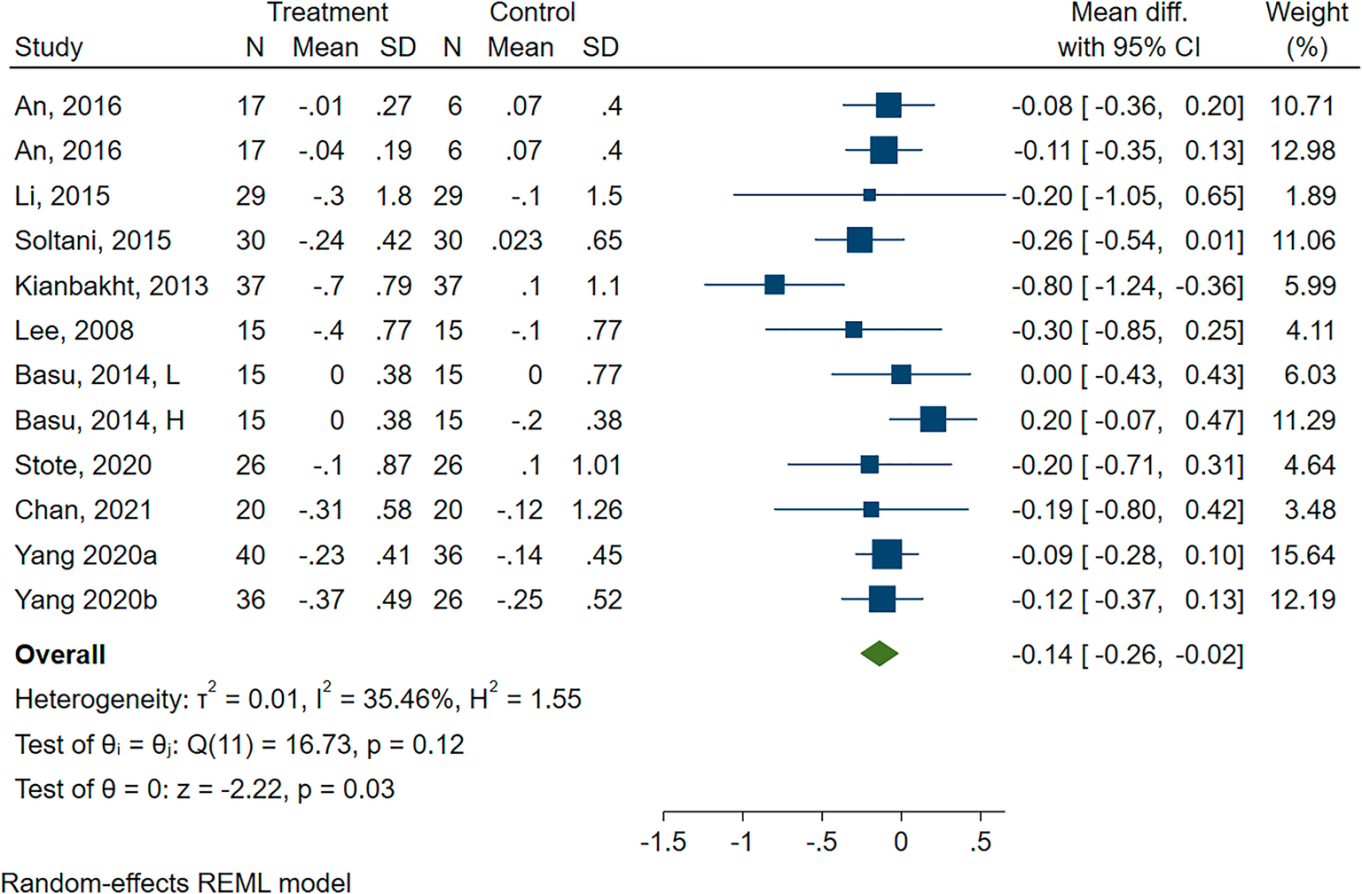
Forest plot of subgroup analysis of the effect of anthocyanins compared with control on HbA1c (%)

There were ten studies (three paired-arms from two studies) that had examined the effects of ACN intake on HbA1c. Meta-analysis results indicated that ACN consumption decreased HbA1c (MD: − 0.14%; 95% CI − 0.26, − 0.02%; P = 0.03). However, subgroup analysis showed this effect only in the participants with T2D (MD: − 0.29%; 95% CI − 0.47, − 0.10%; P = 0.003) and in those trials that used ACN as supplement/extract (MD: − 0.17%; 95% CI − 0.27, − 0.07; P = 0.001) (Fig. 4, and Additional file 1: Figure S14).

The univariate meta-regression suggested that a higher baseline FBG was associated with a better effect of ACN intake on HbA1c changes (β = − 0.13; 95% CI − 0.24, − 0.03; p = 0.011) and this association remained significant after adjusting for ACN dose and baseline BMI (β = − 0.16; 95% CI − 0.31, − 0.019; p = 0.026).

Sensitivity analyses were performed to test the robustness of the results and revealed no remarkable change in overall results (MD: − 0.19%, 95%CI − 0.4, − 0.02%, P = 0.08). The Egger’s tests indicated no significant publication bias (P = 0.532).

The results of leave-one-out analysis were shown in the (Additional file 1: Figure S15).

#### Effect of anthocyanin on serum triglycerides concentrations

We pooled and analyzed data from 38 RCTs evaluating the effects of ACN intake on serum TG concentration. From Four studies [30, 32, 37, 59] that had multi-arm intervention, two pairs of arms were included in the analysis (Additional file 1: Figure S16). The pooled MD was − 9.46 mg/dL (95%CI, − 14.6, − 4.31 mg/dL; P < 0.001, indicating a significantly greater reduction in TG concentrations in participants who received ACN than those used placebo.

The subgroup analysis test showed that there was a significant effect of ACN on TG concentrations in all subgroups of participants (with vs. without dyslipidemia at baseline) and intervention type (supplement/extract vs. food). Furthermore, there were no significant differences between subgroups (p = 0.27 and p = 0.13, respectively) (Additional file 1: Figure S17).

In meta-regression, changes in serum TG concentrations following ACN intervention were found to be independent of treatment duration (β = − 0.49; 95% CI − 2.17, 1.18; p = 0.566), baseline BMI (β = − 0.56; 95% CI − 2.3, 1.18; p = 528) and age of subjects (β = 0.59; 95% CI − 0.39, 1.58; p = 0.239). Nevertheless, changes in TG were significantly associated with the baseline TG concentrations (β = − 0.21; 95% CI − 0.29, − 0.12; p < 0.001).

Results of sensitivity analysis showed significant effect of ACN intake on serum TG similar to the overall effect (MD: − 11.68 mg/dL; 95% CI − 18.04, − 5.32 mg/dL; P < 0.001). The regression asymmetry test was not significant (p = 0.971).

The results of leave-one-out analysis were shown in the (Additional file 1: Figure S18).

#### Effect of anthocyanin on total cholesterol concentrations

Data from 40 trials (four trials with multi-arm interventions) involving 2433 subjects showed that consumption of ACN, as compared with placebo, reduces serum TC concentration (MD: − 6.98%; 95% CI − 11.97, − 2.0 mg/dL; P = 0.01) (Additional file 1: Figure S19).

Subgroup analyses were carried out for participants (with vs. without dyslipidemia at baseline) and intervention (supplement/extract vs. food). The mean change in TC concentration in trials using supplement/extract (MD: − 9.76 mg/dL, 95% CI − 17.33 to − 2.2 mg/dL; P = 0.011) was greater than from daily foods (MD: − 3.10 mg/dL; 95% CI − 8.24, 2.05 mg/dL; P = 0.238). Also, studies with baseline dyslipidemia showed greater decreases in TC concentrations (MD: − 13.41 mg/dL; 95% CI − 22.95, − 3.88 mg/dL; P = 0.006) than those without baseline dyslipidemia (MD: − 2.30 mg/dL; 95% CI − 6.78, 2.18 mg/dL; P = 0.315) (Additional file 1: Figure S20).

The univariate meta-regression suggested that a higher baseline TC was associated with a better effect of ACN intake on TC changes (β = − 0.36; 95% CI − 0.60, − 0.15; P = 0.002). Adjustments for ACN dose (β = 0.017; 95% CI − 0.02, 0.052; P = 0.327), duration of intervention (β = 1.12; 95% CI − 0.19, 1.44; P = 0.853) and baseline BMI (β = − 0.36; 95% CI − 1.71, 0.98; P = 0.594) revealed no significant effect of these variables.

Sensitivity analyses were performed to test the robustness of the results and showed effects similar to the overall effects (MD: − 8.63 mg/dL, 95%CI − 14.6, − 2.61, P < 0.001). The regression asymmetry tests suggested that no significant publication bias (P = 0.200).

The results of leave-one-out analysis were shown in supplementary file (Additional file 1: Figure S21).

#### Effect of anthocyanin on LDL-C concentrations

Thirty nine trials compared the effect of ACN intervention on serum LDL-C concentrations with control. Pooled data showed statistically significant effect on LDL-C changes (MD: − 6.91 mg/dL; 95% CI − 9.99, − 3.83 mg/dL; P < 0.001) (Additional file 1: Figure S22).

The effect of ACN was similar in all types of intervention (supplement/extract vs. food) (Additional file 1: Figure S23). Nevertheless, ACN intake could decrease serum LDL-C concentrations only in dyslipidemic subjects (MD: − 10.68 mg/dL; 95% CI − 14.85, − 6.52 mg/dL; P < 0.001) but not in those with normol blood lipids (MD: − 3.77 mg/dL; 95% CI − 7.6, 0.06: P = 0.054).

Our meta-regression suggested the association between ACN intake and LDL-C was not affected by duration of intrvention (β = − 0.25; 95% CI − 1.03, 0.53; P = 0.531), dose of intervention (β = − 0.003; 95% CI − 0.028, 0.021; P = 0.786), BMI at baseline (β = − 0.56; 95% CI − 1.54, 0.41; P = 0.257) and LDL-C concentrations at baseline (β = − 0.12; 95% CI − 0.32, 0.07; P = 0.216).

Results of sensitivity analysis showed significant effect of ACN intake on serum LDL-C MD:—7.72 mg/dL; 95% CI − 11.23, − 4.21 mg/dL; P < 0.001). The regression asymmetry test was not significant (p = 0.365).

The results of leave-one-out analysis were shown in (Additional file 1: Figure S24).

#### Effect of anthocyanin on serum HDL-C concentrations

Serum HDL-C concentration was evaluated in 39 studies (four studies with multiple-arms). Meta-analysis revealed positive effect of ACN intake on HDL-C concentrations (MD: 1.71 mg/dL; 95% CI 0.43, 2.08 mg/dL; p = 0.01) (Additional file 1: Figure S25).

Subgroup analyses were carried out for participants (with vs. without dyslipidemia at baseline) and intervention (supplement/extract vs. food). The mean change in serum HDL-C concentration in those trials on subjects with dyslipidemia (MD: 2.35 mg/dL; 95% CI 0.26, 4.45 mg/dL; P = 0.028) was greater than the trials conducted on participants normal blood lipids (MD: 0.73 mg/dL; 95% CI 0.03, 1.43 mg/dL; P = 0.041) (Additional file 1: Figure S26).

Findings from meta-regression suggested the association between ACN intake and serum HDL-C was not affected by duration of intervention (β = 0.26; 95% CI − 0.06, 0.58; P = 0.114), dose of intervention (β = − 0.002; 95% CI − 0.01, 0.005; P = 0.494), BMI at baseline (β = − 0.0008; 95% CI − 0.34, 0.34; P = 0.996) and serum HDL-C concentrations at baseline (β = − 0.04; 95% CI − 0.09, 0.012; P = 0.130).

Sensitivity analyses were performed to test the robustness of the results and showed positive effect of ACN intake on HDL-C concentrations similar to the overall effects (MD: − 2.16 mg/dL, 95% CI 0.71, 3.6 mg/dL, P < 0.001). The Egger’s tests indicated no significant publication bias (P = 0.182).

The results of leave-one-out analysis were shown in (Additional file 1: Figure S27).

#### Effect of anthocyanin on serum apolipoprotein A (Apo A) concentrations

Meta-analysis of RCTs yielded a weighted estimate for the overall effect of ACN intake of MD: 3.94 mg/dL (95% CI 0.77 to 7.11) on Apo A concentrations (Additional file 1: Fig. S28). Apo A estimates were not significantly different among subgroups (without dyslipidemia vs. with dyslipidemia and supplement/extract vs. food) (Additional file 1: Figure S29).

The meta-regression analysis suggested that effect of the ACN intake on Apo A concentrations was not affected by duration of intrvention (β = − 0.49; 95% CI − 2.5, 1.51; P = 0.631), dose of intervention (β = 0.0005; 95% CI − 0.02, 0.021; P = 0.962), BMI at baseline (β = − 0.71; 95% CI − 2.76, 1.32; P = 0.490) and Apo A concentrations at baseline (β = 0.18; 95% CI − 0.06, 0.42; P = 0.155).

Results of sensitivity analysis showed similar significant effect of ACN intake on serum apo A, (MD: 3.91 mg/dL; 95% CI 0.16, 7.65 mg/dL; P = 0.04). The regression asymmetry test was not significant (p = 0.301).

The results of leave-one-out analysis were shown in (Additional file 1: Figure S30).

#### Effect of anthocyanin on serum apolipoprotein B (Apo B) concentrations

The meta-analysis showed that ACN intake was not associated with significant changes in serum apo B concentrations (MD: − 2.36 mg/dL; 95% CI − 5.55, 0.83 mg/dL; P = 0.15). There was no statistically significant changes in circulating apo B in subgroup analysis, either (Additional file 1: Figures S31 and S32).

The meta-regression analysis suggested that effect of ACN intake on serum apo B concentrations was not affected by duration of intervention (β = − 1.13; 95% CI − 3.64, 1.38; P = 0.378), dose of intervention (β = − 0.015; 95% CI − 0.04, 0.009; P = 0.223), BMI at baseline (β = 3.6; 95% CI − 3.02, 10.22; P = 0.287) and apo B concentrations at baseline (β = 0.39; 95% CI − 0.26, 1.05; P = 0.241).

Results of sensitivity analysis showed no significant effect of ACN intake on serum apo B, (MD: − 1.9 mg/dL; 95% CI − 5.27, 1.47 mg/dL; P = 0.27). The regression asymmetry test was not significant, either (p = 0.128).

The results of leave-one-out analysis were shown in (Additional file 1: Figure S33).

## Discussion

The findings of this meta-analysis confirmed the positive effects of ACN intake on certain cardiometabolic risk factors including BMI and more importantly FM, glycemic and lipidemic profiles. A combination of these factors occur in metabolic syndrome (MetS) that is characterized by central obesity, dysglycemia, above normal blood pressure, low serum HDL-C and high TG concentrations [69]. There is a remarkable higher risk of developing various diseases including CVD and diabetes in the subjects with MetS and that is why prevention and treatment of MetS can actually reduce CVD risk [70].

### Anthocyanins and body mass index

We found the positive effect of ACN consumption on reduction of BMI which further confirms previous reports [71, 72]. Nevertheless, our finding on the supplement form-dependent effect of ACN consumption on BMI is quite noticeable, i.e. ACN supplement (like tablet) causes decrease whereas powdered ACN results in increase in BMI. On re-analysis of the data, however, we found out that this controversial finding was mostly due to one study [41]. Notwithstanding, in that study the increase in body weight and hence BMI was mostly due to lean body mass and not fat mass [41]. When we excluded this study from analysis, this effect was removed (MD − 0.14 (− 0.66, 0.9), P = 0.610).

Sensitivity analysis using the leave-one-out approach indicated that the direction of the combined estimates did not vary markedly with the removal of each study in turn. However, removal of Stull et al. [41] that used yogurt and skim milk-based smoothie as intervention, changed the effect size from − 0.21 to − 0.35.

#### Anthocyanins and body fat mass

Some evidence indicates that truncal adiposity is the core constituent of the metabolic derrangements observed in MetS [73, 74]. Our data showed the positive effect of ACN intake on total FM and not on central obesity, as judged by WC. However, we were unable to perform subgroub analysis due to the limited eligible RCTs.

The association between body FM variability and CVD risk has already been reported [75]. Nonetheless, strong evidence supports the contribution of truncal fat distribution, even in the context of normal BMI, to the increased risk of CVD [76, 77]. One possible explanation for this dicrepancy in results could be the difference in precision of the methods of two assessements as FM is assessed using an instrument like dual-energy X-ray absorptiometry (DEXA) whereas WC is measured using a measuring tape that is less sensitive to detect changes. This is especially the case when changes occur in visceral fat [78] that is more related to CVD risk [79].

The exact mechanism of anti-adipogenesis function of ACN has not been fully elucidated yet. Some evidence suggests an inhibitory effect of ACN against adipocyte differentiation that might be exerted through 5' adenosine monophosphate-activated protein kinase (AMPK) signaling pathway activation [80, 81].

#### Anthocyanins and glycemic status

The findings of this meta-analysis confirmed the alleviative effect of ACN on glycemic status including FBS and HbA1c. Notwithstanding, the effect on both variables was more prominent in those subjects with above-normal values, notably in the subjects with T2D. It has been documented that ACN has the potential to upregulate and also downregulate certain glucose metabolic pathways including AMPK whereby exerts its modulating effect on glucose homeostasis [82]. Other proposed mechanisms for anti-diabetic actions of ACN include inhibition of α-amylase, α-glucosidase and modulating effect on glucose transporters, inhibition of glycogenolysis and modulation of gut microbiota [83].

We found better effect on HbA1c of ACN when consumed as a supplement. It has been shown that ACN in foods may interact with other biomolecules including proteins and polysaccharides that may affect ACN activity [83]. Evaluation of the efficacy of ACN in natural foods versus supplements need further investigations.

#### Anthocyanins and lipidemic status

Our findings documented the effects of ACN on healthy changes of blood lipids including a decrement of TG, TC and LDL-C and an increment of HDL-C. Again here, better effects were achieved in the subjects with deranged blood lipid profile. Nevertheless, data from this meta-analysis failed to show any effect of ACN on serum concentrations of apo A and apo B. The mechanism of action of ACN on healthy modification of blood lipids is under investigation. Some studies indicate that ACN can reduce TC possibly via increased fecal excretion of neutral and acidic sterols together with down-regulation of hepatic β-hydroxy β-methyl glutaryl coenzyme A (HMGCoA) reductase, the rate-limiting enzyme of cholesterol biosynthesis [84]. The mechanism of TG-lowering effect of ACN remains to be elucidated. This effect has been attributed to the reduction of apo B and CIII [85]. Notwithstanding, our meta-analysis failed to show any significant effect of ACN on serum apo B concentrations. ACN-induced decrement of circulating LDL-C may be partly due to downregulation of cholesteryl ester transfer protein [86] and upregulation of LDL-receptor [87]. It has also been proposed that ACN can affect both liver and blood lipids contents via activation of AMPK. Activated AMPK in turn induces acetyl coenzyme A carboxylase phosphorylation and upregulates hepatic peroxisome proliferator-activated receptor-alpha (PPAR-α), acylCoA oxidase and palmitoyltrasferase-1 A [88].

## Limitations

This study has some limitations. We did not evaluate the antioxidant effect of ACN that can have a major contribution to prevention of CVD [89, 90] and other diseases [91, 92]. Nonetheless, the efficacy of ACN as an antioxidant has been recently confirmed in a meta-analysis [93]. Limited studies on effects of ACN intake on waist circumference, fat mass, apo A and apo B concentrations (n < 10) could effect on generalizability of results of meta-regression tests. Furthermore, we did not evaluate the effect of ACN on biomarkers of systemic and vascular inflammation, which has an axial role in both development and progression of CVD [94, 95].

The other limitation of our study is scanty available RCTs that evaluated the long-term effects of ACN intake. Though some RCTs had treatment durations of up to two years, the question of the effectiveness of long-term ACN intake remains unanswered due to the lack of experimental evidence.

In some RCTs, especially those that used ACN-containing foods, the exact amount of ACN consumption was not reported. Tt was not, therefore, possible to perform dose–response analysis. Besides, in most RCTs, ACN intake from dietary sources was not adjusted and this could affect the results.

The intervention strategy across the RCTs was diverse with respect to the dosage of ACN, the frequency of intake and type of intervention (food/supplement), which inevitably contributed to the high levels of heterogeneity. We attempted to reduce the heterogeneity by choosing appropriate statistical models and using sensitivity analyses. Notwithstanding, this point should be considered when interpreting and generalizing the results.

Finally, the present meta-analysis employed just the papers published in English. Thus, the papers in other languages or unpublished data might potentially increase the heterogeneity in the present results.

## Conclusion

The findings of this meta-analysis indicate that ACN intake in the forms of natural foods and supplements can induce healthy changes in body FM, glycemic and lipidemic status and these effects are more prominent in the subjects with above-normal values. Further well-designed studies are needed to evaluate and compare the efficacy of ACN consumption as natural food sources versus supplements. Meanwhile, fortification of foods and drinks with ACN can also be considered to increase ACN intake in the general population as a preventive strategy against many diseases notably CVD.

## Supplementary Information

**Additional file 1**: **Figure 1S**. Risk of bias summary: Reviewer authors’ judgements about each “risk of bias” item for every single included study; **Figure 2S**. Risk of bias graph: review authors' judgments about each risk of bias item presented as percentages across all included studies; **Figure 3S**. Forest plot of the effect of anthocyanins compared with control on body mass index (kg/m2); **Figure 4S**. The result of leave-one-out analysis of the effect of anthocyanin on body mass index (kg/m2); **Figure 5S**. Forest plot of the effect of anthocyanins compared with control on waist circumference (cm); **Figure 6S**. The result of leave-one-out analysis of the effect of anthocyanin on waist circumference (cm); **Figure 7S**. Forest plot of the effect of anthocyanins compared with control on body fat mass (%); **Figure 8S**. The result of leave-one-out analysis of the effect of anthocyanin on body fat mass (%); **Figure 9S**. Forest plot of the effect of anthocyanins compared with control on fasting blood glucose (mg/dL); **Figure 10S**. The result of leave-one-out analysis of the effect of anthocyanin on fasting blood glucose (mg/dL); **Figure 11S**. Forest plot of the effect of anthocyanins compared with control on fasting serum insulin (µIU/mL); **Figure 12S**. Forest plot of subgroup analysis of the effect of anthocyanins compared with control on fasting serum insulin (µIU/mL); **Figure 13S**. The result of leave-one-out analysis of the effect of anthocyanin on fasting serum insulin (µIU/mL); **Figure 14S**. Result of subgroup analysis of the effect of anthocyanins compared with control on HbA1c (%); **Figure 15S**. The result of leave-one-out analysis of the effect of anthocyanin on HbA1c (%); **Figure 16S**. Forest plot of the effect of anthocyanins compared with control on serum triglycerides concentrations (mg/dL); **Figure 17S**. Result of subgroup analysis of the effect of anthocyanins compared with control on serum triglyceride concentrations (mg/dL); **Figure 18S**. The result of leave-one-out analysis of the effect of anthocyanin on serum triglyceride concentrations (mg/dL); **Figure 19S**. Forest plot of the effect of anthocyanins compared with control on serum total cholesterol (mg/dL); **Figure 20S**. Result of subgroup analysis of the effect of anthocyanins compared with control on serum total cholesterol (mg/dL); **Figure 21S**. The result of leave-one-out analysis of the effect of anthocyanin on serum total cholesterol concentrations (mg/dL); **Figure 22S**. Forest plot of the effect of anthocyanins compared with control on serum LDL-C concentrations (mg/dL); **Figure 23S**. The result of subgroup analysis of the effect of anthocyanins compared with control on serum LDL-C concentrations (mg/dL); **Figure 24S**. The result of leave-one-out analysis of the effect of anthocyanin on serum LDL-C concentrations (mg/dL); **Figure 25S**. Forest plot of the effect of anthocyanins compared with control on serum HDL-C concentrations (mg/dL); **Figure 26S**. Result of subgroup analysis of the effect of anthocyanins compared with control on serum HDL-C concentrations (mg/dL); **Figure 27S**. The result of leave-one-out analysis of the effect of anthocyanin on serum HDL-C concentrations (mg/dL); **Figure 28S**. Forest plot of the effect of anthocyanins compared with control on serum Apo A concentrations (mg/dL); **Figure 29S**. Result of subgroup analysis of the effect of anthocyanins intake compared with control on serum Apo A concentrations (mg/dL); **Figure 30S**. The result of leave-one-out analysis of the effect of anthocyanin on serum Apo A concentrations (mg/dL); **Figure 31S**. Forest plot of the effect of anthocyanins compared with control on serum Apo B concentrations (mg/dL); **Figure 32S**. Result of subgroup analysis of the effect of anthocyanins intake compared with control on serum Apo B concentrations (mg/dL); **Figure 33S**. The result of leave-one-out analysis of the effect of anthocyanin on serum Apo B concentrations (mg/dL).

## Data Availability

All data generated or analyzed during this study were presented in this article.

## Abbreviations

DEXA: Dual-energy X-ray absorptiometry
FBG: Fasting blood glucose
FM: Fat mass
FSI: Fasting serum insulin
HbA1c: Glycated hemoglobin
HDL-C: High density lipoprotein-cholesterol
HMGCoA: β-Hydroxy β-methyl glutaryl coenzyme A (HMGCoA)
HOMA-IR: Homeostasis model assessment-estimated insulin resistance
LDL-C: Low density lipoprotein-cholesterol (LDL-C)
MD: Mean difference
MetS: Metabolic syndrome
PPAR-α: Peroxisome proliferator-activated receptor-alpha
RCTs: Randomized clinical trials
TC: Total cholesterol
T2D: Type 2 diabetes
TG: Triglyceride
WC: Waist circumference
WHO: World Health Organization
WoS: Web of Science
