# Supplementary material for: How effective are anthocyanins on healthy modification of cardiometabolic risk factors: a systematic review and meta-analysis
Source: Diabetol Metab Syndr. 2023 May 23;15:106. doi: 10.1186/s13098-023-01075-0 (PMC10204221; doi:10.1186/s13098-023-01075-0)
Supplement: Supplementary file 1 — Additional file 1: Figure 1S. Risk of bias summary: Reviewer authors’ judgements about each “risk of bias” item for every single included study; Figure 2S. Risk of bias graph: review authors' judgments about each risk of bias item presented as percentages across all included studies; Figure 3S. Forest plot of the effect of anthocyanins compared with control on body mass index (kg/m2); Figure 4S. The result of leave-one-out analysis of the effect of anthocyanin on body mass index (kg/m2); Figure 5S. Forest plot of the effect of anthocyanins compared with control on waist circumference (cm); Figure 6S. The result of leave-one-out analysis of the effect of anthocyanin on waist circumference (cm); Figure 7S. Forest plot of the effect of anthocyanins compared with control on body fat mass (%); Figure 8S. The result of leave-one-out analysis of the effect of anthocyanin on body fat mass (%); Figure 9S. Forest plot of the effect of anthocyanins compared with control on fasting blood glucose (mg/dL); Figure 10S. The result of leave-one-out analysis of the effect of anthocyanin on fasting blood glucose (mg/dL); Figure 11S. Forest plot of the effect of anthocyanins compared with control on fasting serum insulin (µIU/mL); Figure 12S. Forest plot of subgroup analysis of the effect of anthocyanins compared with control on fasting serum insulin (µIU/mL); Figure 13S. The result of leave-one-out analysis of the effect of anthocyanin on fasting serum insulin (µIU/mL); Figure 14S. Result of subgroup analysis of the effect of anthocyanins compared with control on HbA1c (%); Figure 15S. The result of leave-one-out analysis of the effect of anthocyanin on HbA1c (%); Figure 16S. Forest plot of the effect of anthocyanins compared with control on serum triglycerides concentrations (mg/dL); Figure 17S. Result of subgroup analysis of the effect of anthocyanins compared with control on serum triglyceride concentrations (mg/dL); Figure 18S. The result of leave-one-out analysis of the effect of [file 13098_2023_1075_MOESM1_ESM.docx]

**How effective are anthocyanins on healthy modification of cardiometabolic risk factors: a systematic review and meta-analysis**

Tirang R. Neyestani, Zahra Yari, Hamid Rasekhi, Bahareh Nikooyeh^*^

**Additional data**


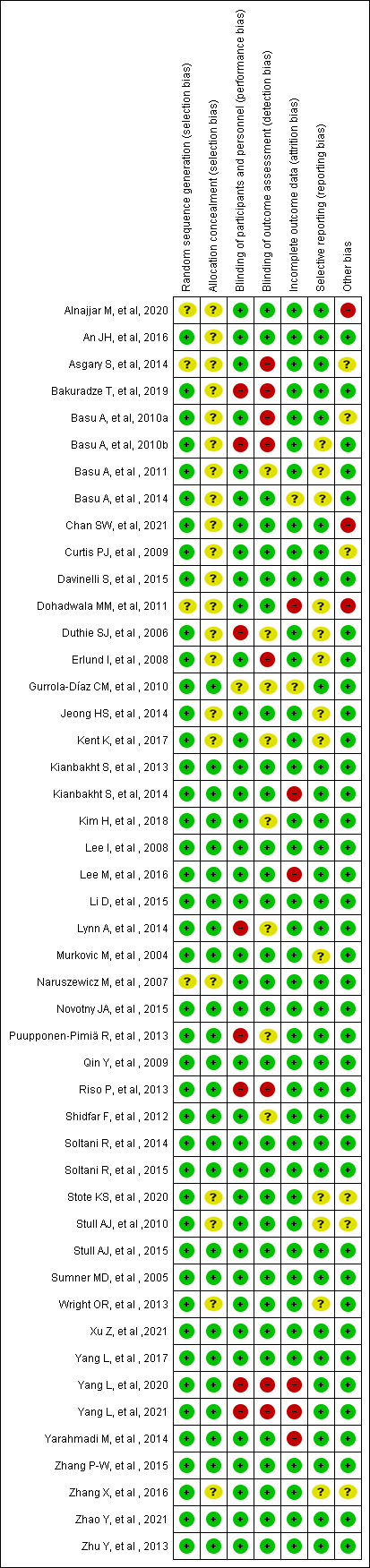


Figure 1S. Risk of bias summary:

Reviewer authors’ judgements about each “risk of bias” item for every single included study.


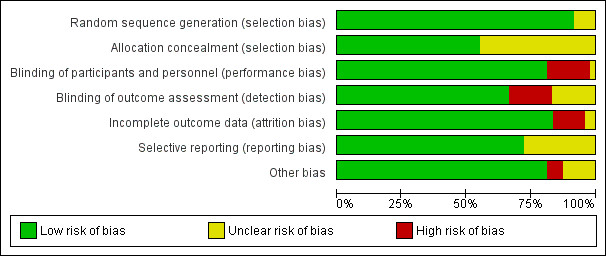


Figure 2S. Risk of bias graph: review authors' judgments about each risk of bias item presented as percentages across all included studies.


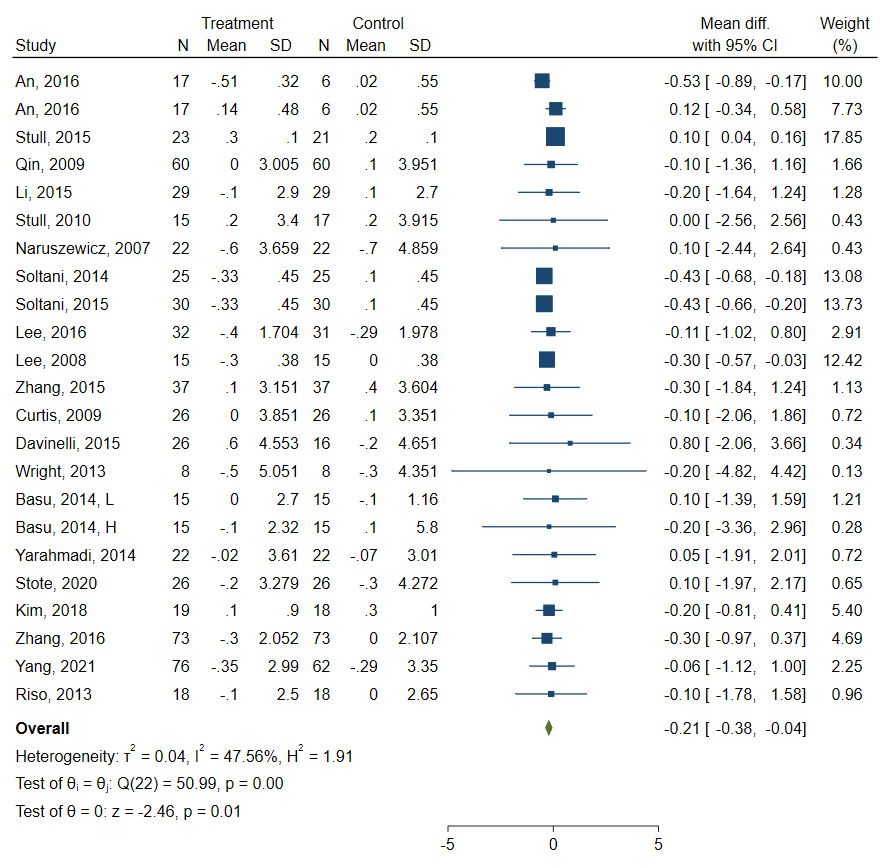


Figure 3S. Forest plot of the effect of anthocyanins compared with control on body mass index (kg/m^2^)


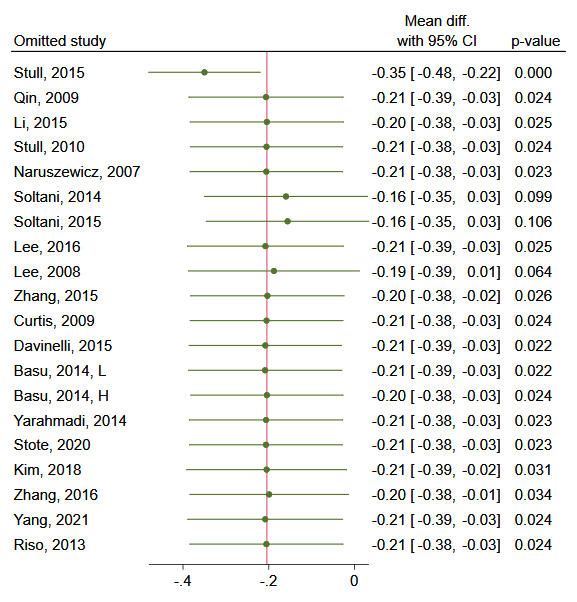


Figure 4S. The result of leave-one-out analysis of the effect of anthocyanin on body mass index (kg/m^2^)


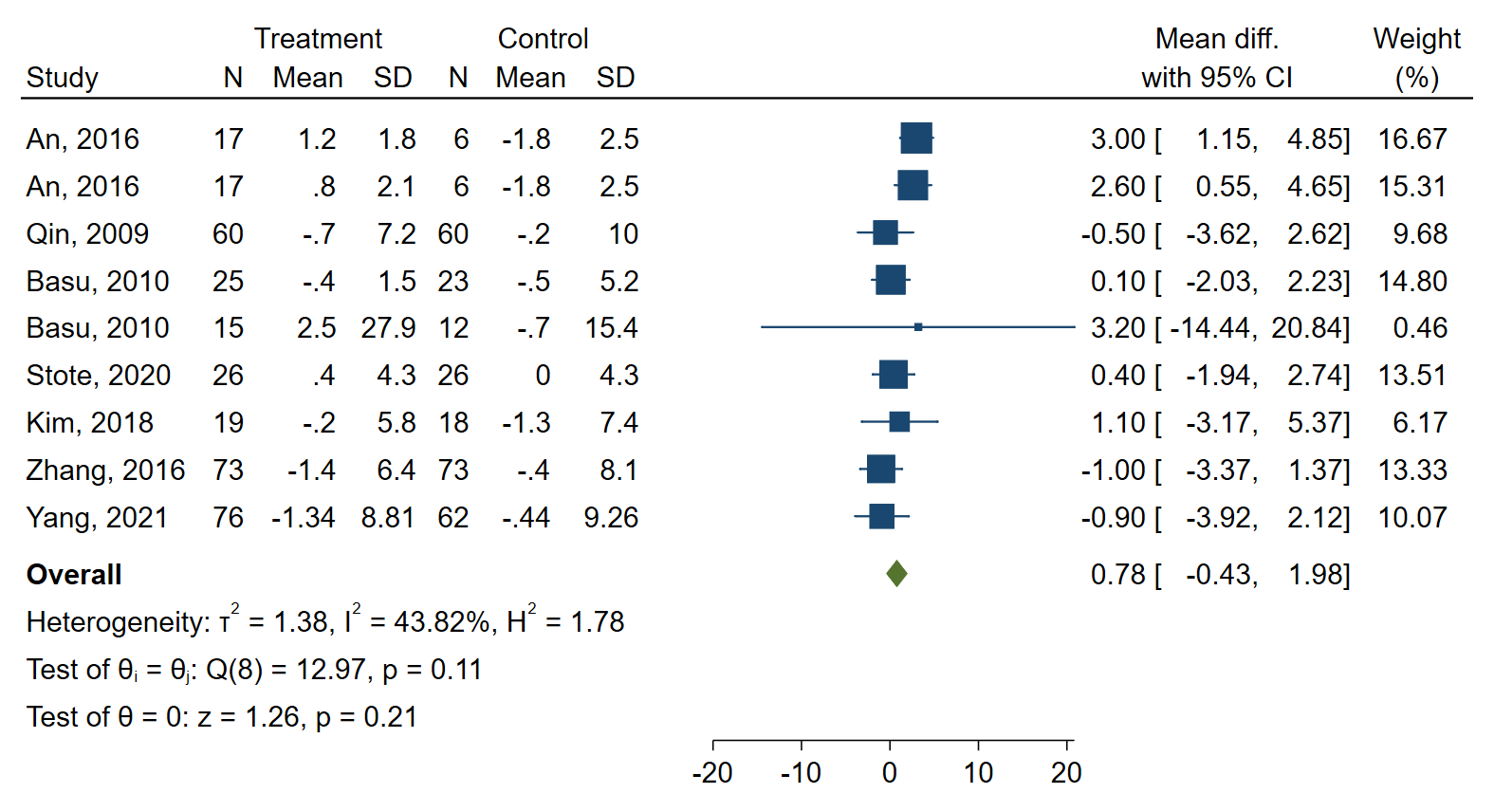


Figure 5S. Forest plot of the effect of anthocyanins compared with control on waist circumference (cm)


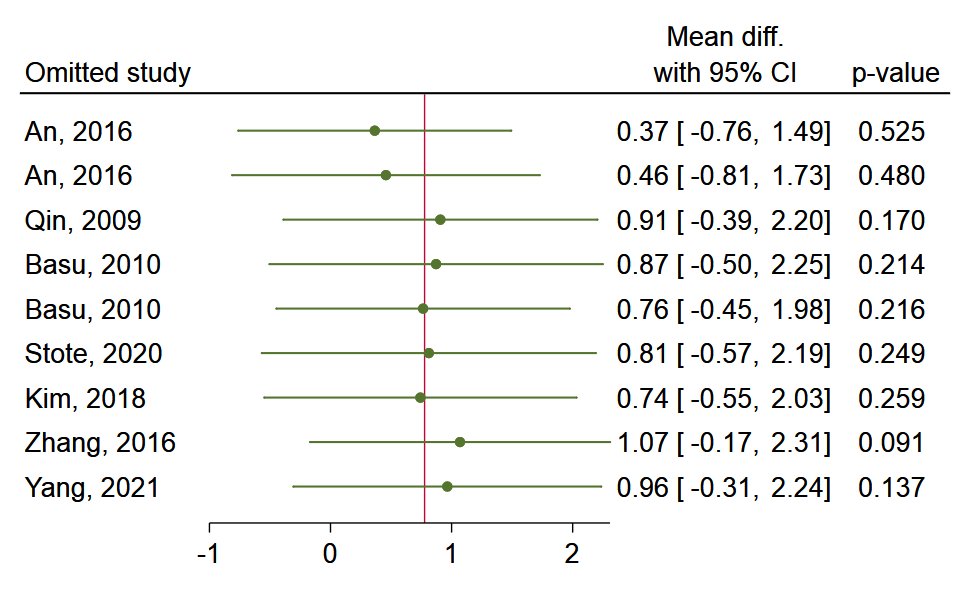


Figure 6S. The result of leave-one-out analysis of the effect of anthocyanin on waist circumference (cm)


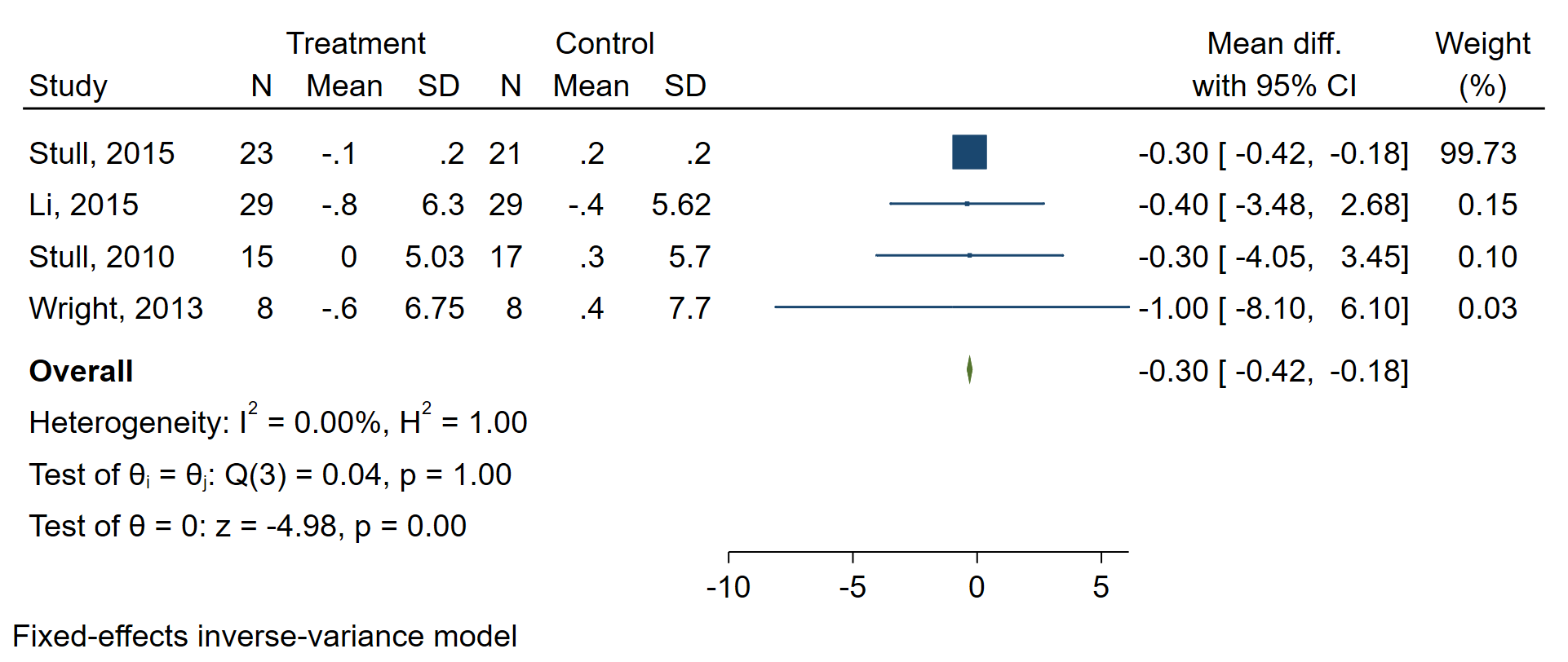


Figure 7S. Forest plot of the effect of anthocyanins compared with control on body fat mass (%)


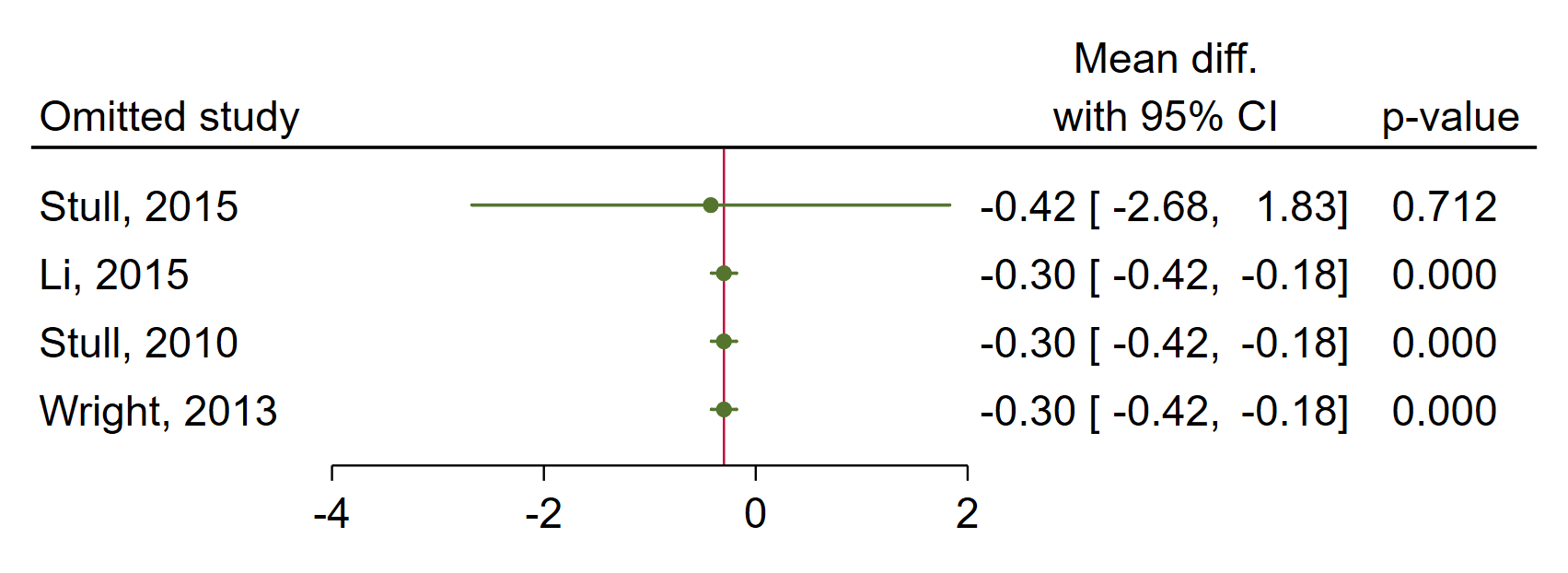


Figure 8S. The result of leave-one-out analysis of the effect of anthocyanin on body fat mass (%)


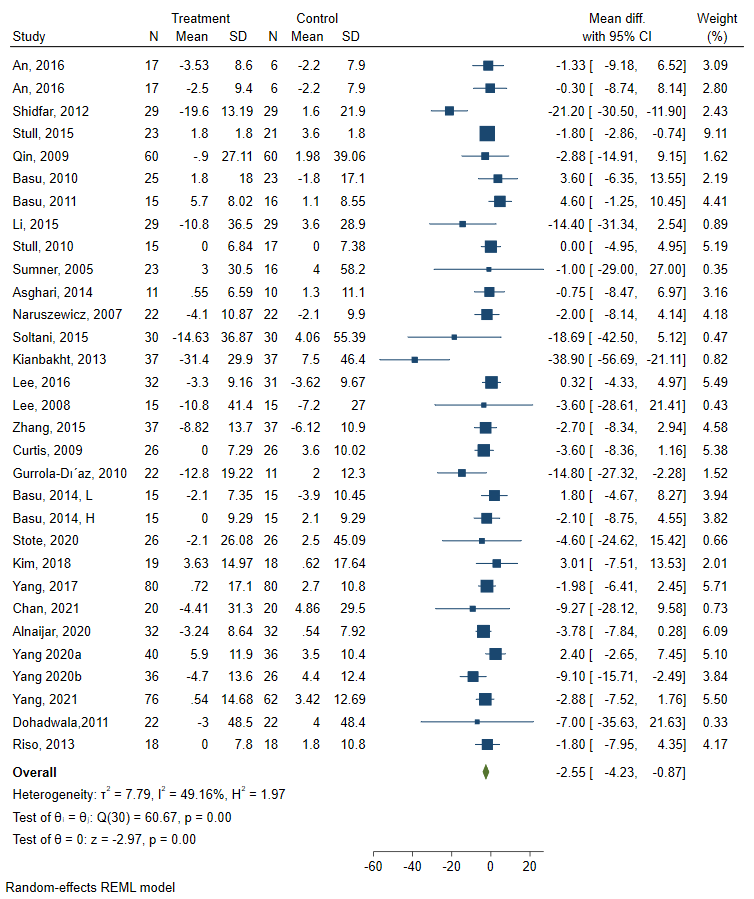


Figure 9S. Forest plot of the effect of anthocyanins compared with control on fasting blood glucose (mg/dL)


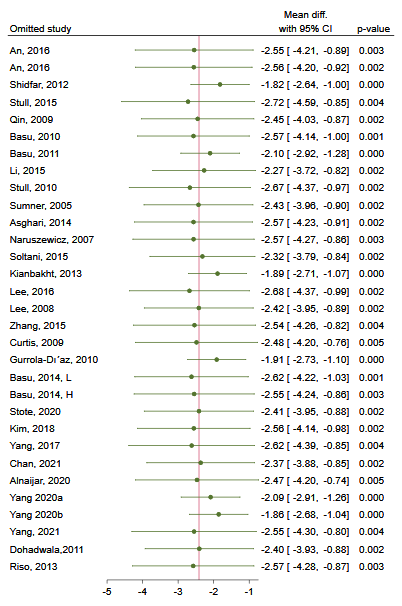


Figure 10S. The result of leave-one-out analysis of the effect of anthocyanin on fasting blood glucose (mg/dL)


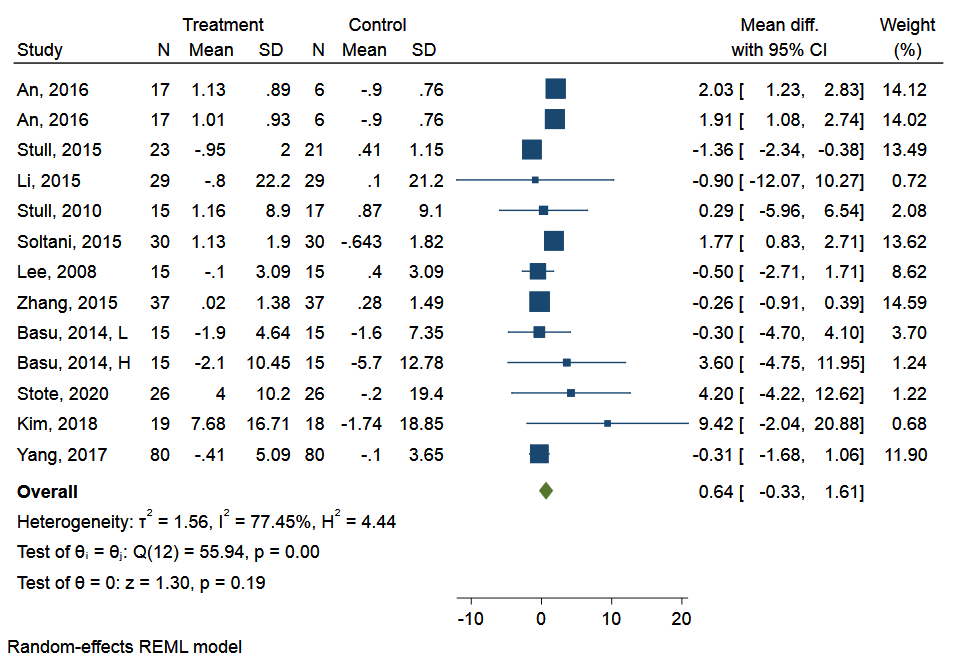


Figure 11S. Forest plot of the effect of anthocyanins compared with control on fasting serum insulin (µIU/mL)


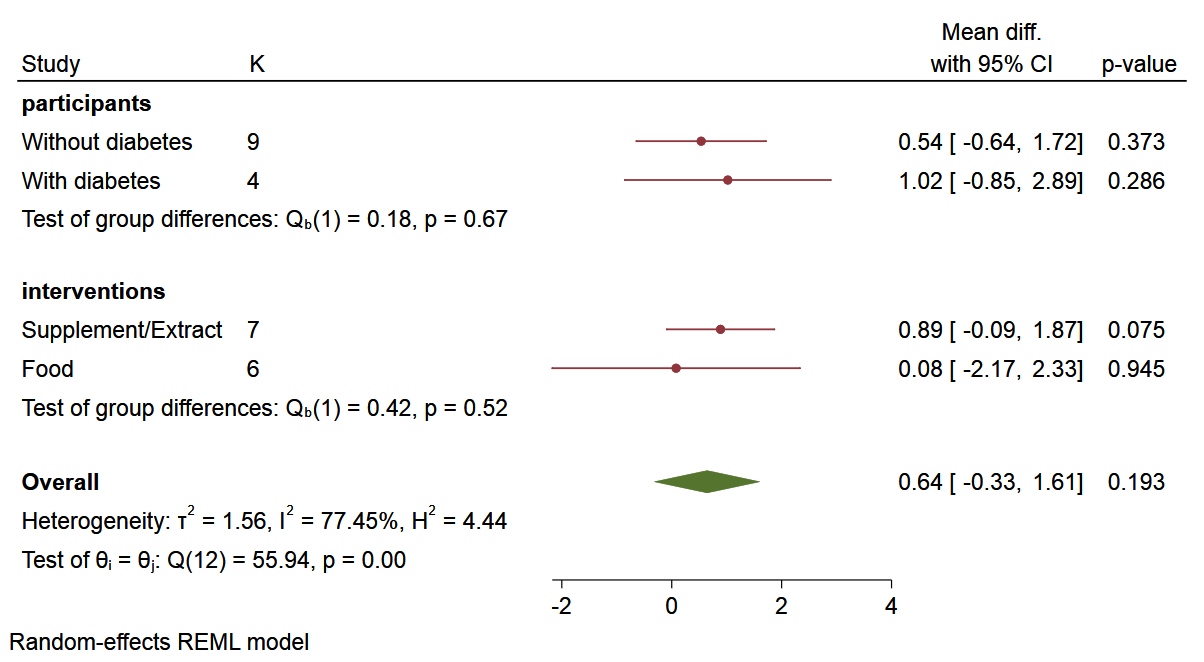


Figure 12S. Forest plot of subgroup analysis of the effect of anthocyanins compared with control on fasting serum insulin (µIU/mL)


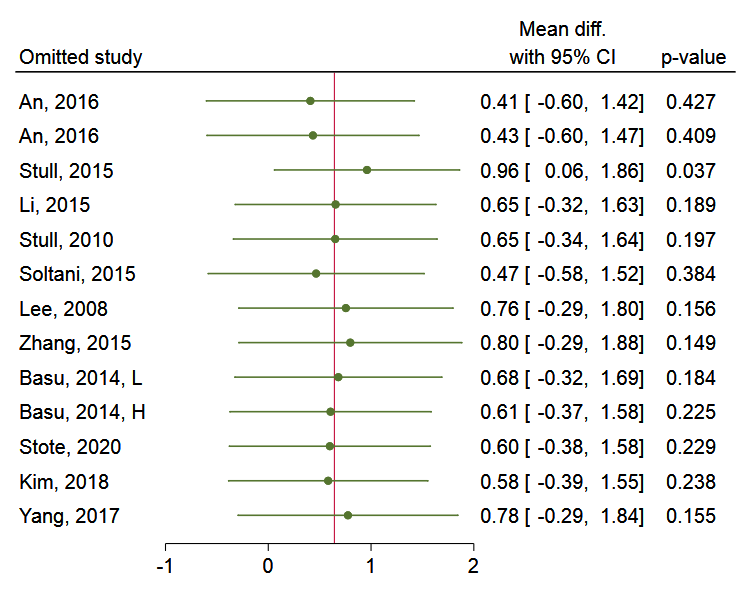


Figure 13S. The result of leave-one-out analysis of the effect of anthocyanin on fasting serum insulin (µIU/mL)


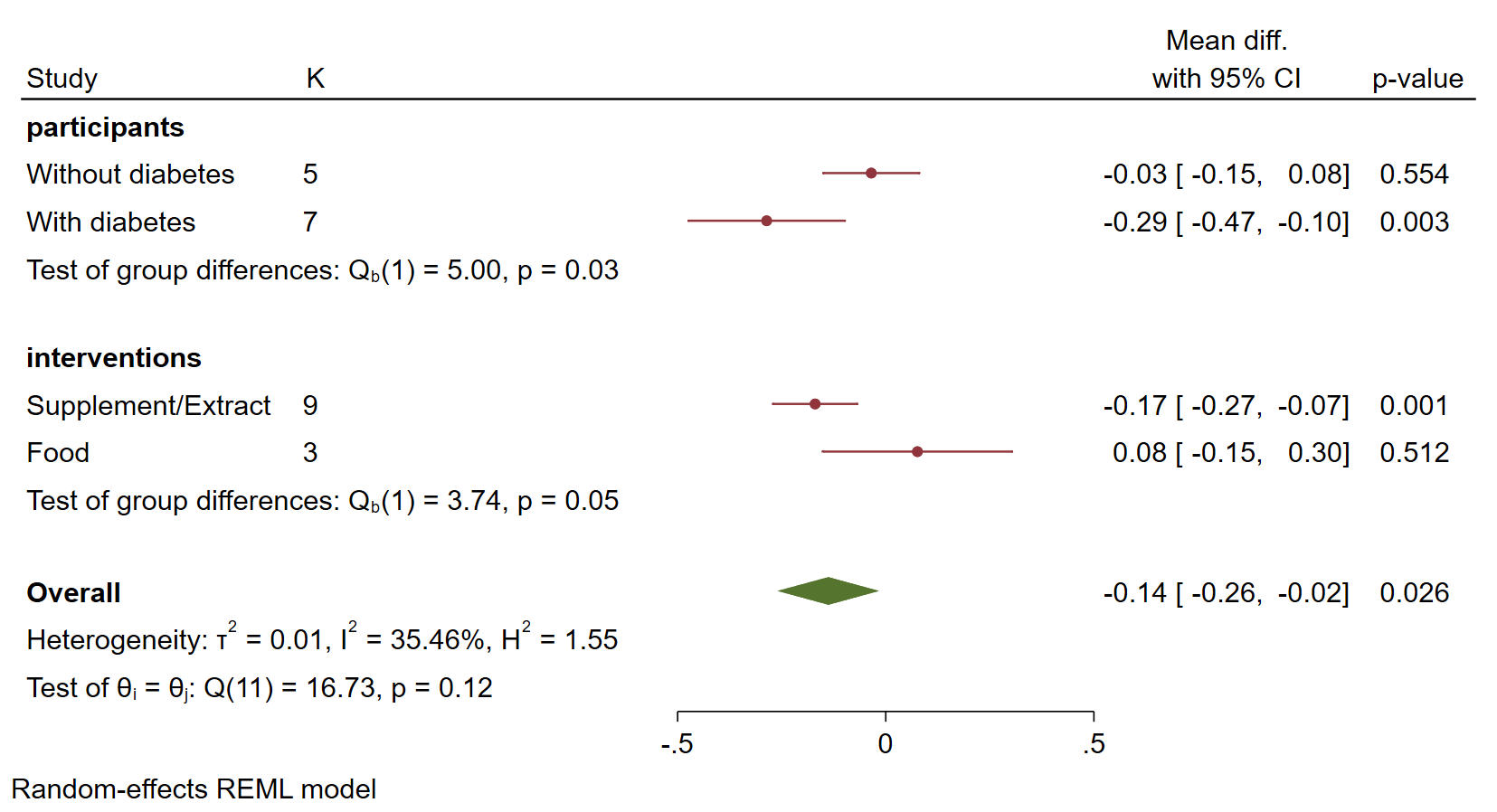


Figure 14S. Result of subgroup analysis of the effect of anthocyanins compared with control on HbA1c (%)


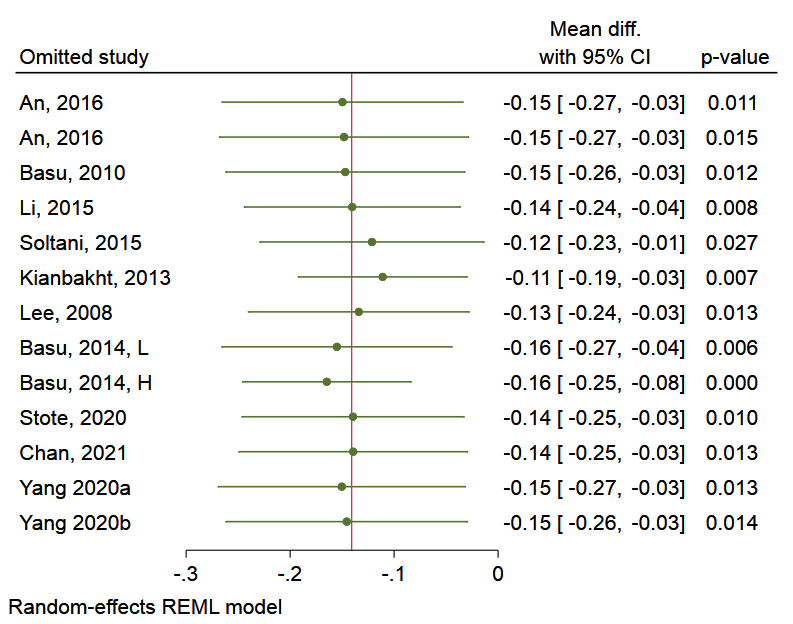


Figure 15S. The result of leave-one-out analysis of the effect of anthocyanin on HbA1c (%)


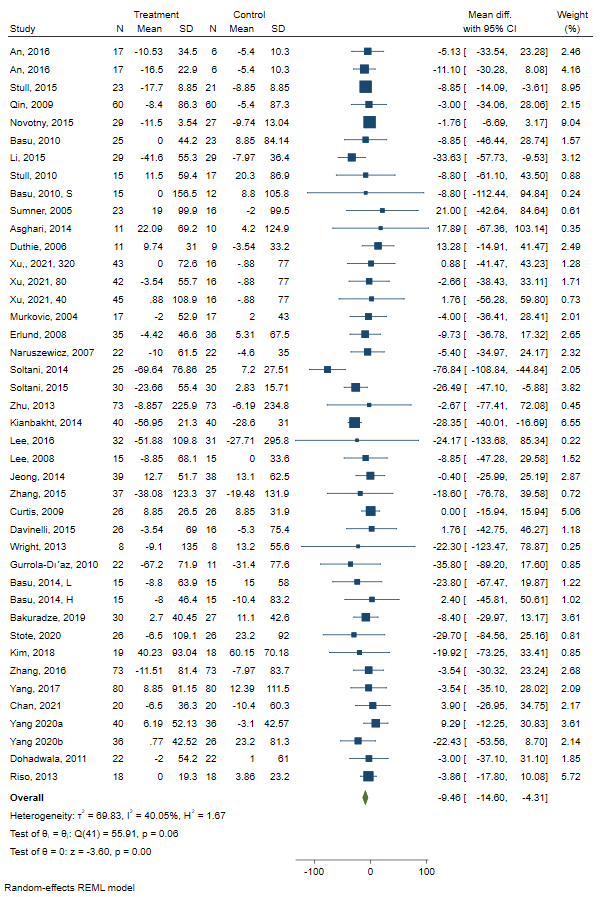


Figure 16S. Forest plot of the effect of anthocyanins compared with control on serum triglycerides concentrations (mg/dL)


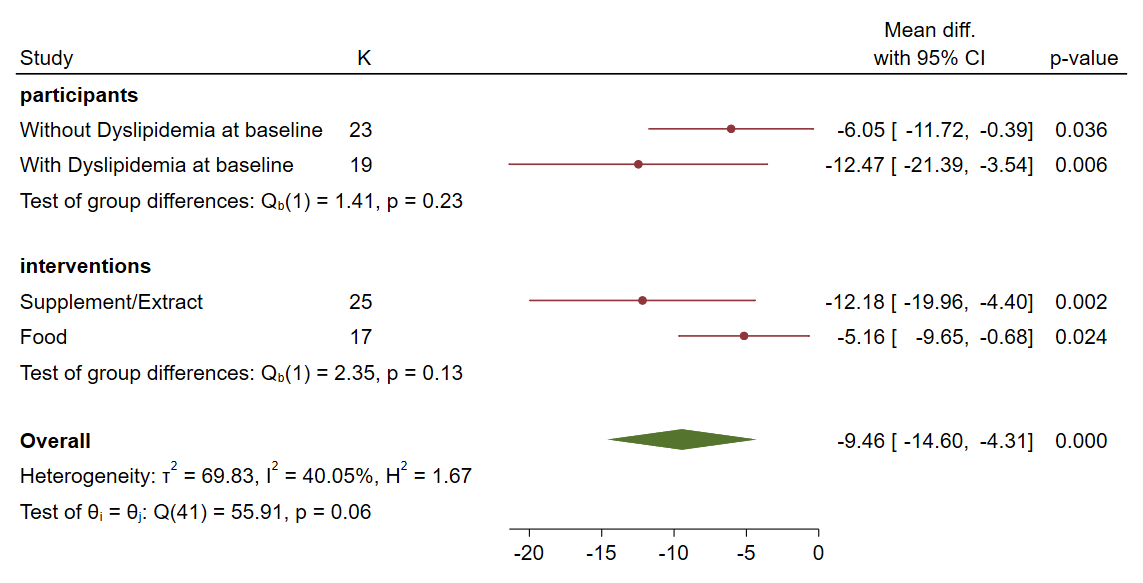


Figure 17S. Result of subgroup analysis of the effect of anthocyanins compared with control on serum triglyceride concentrations (mg/dL)


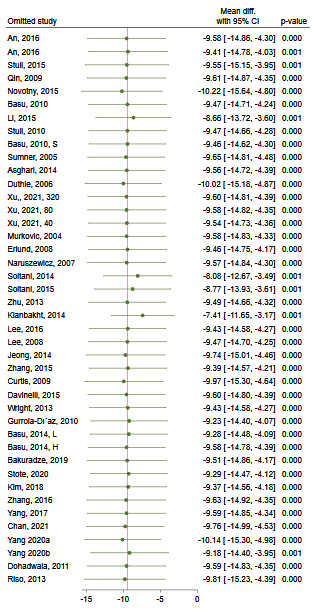


Figure 18S. The result of leave-one-out analysis of the effect of anthocyanin on serum triglyceride concentrations (mg/dL)


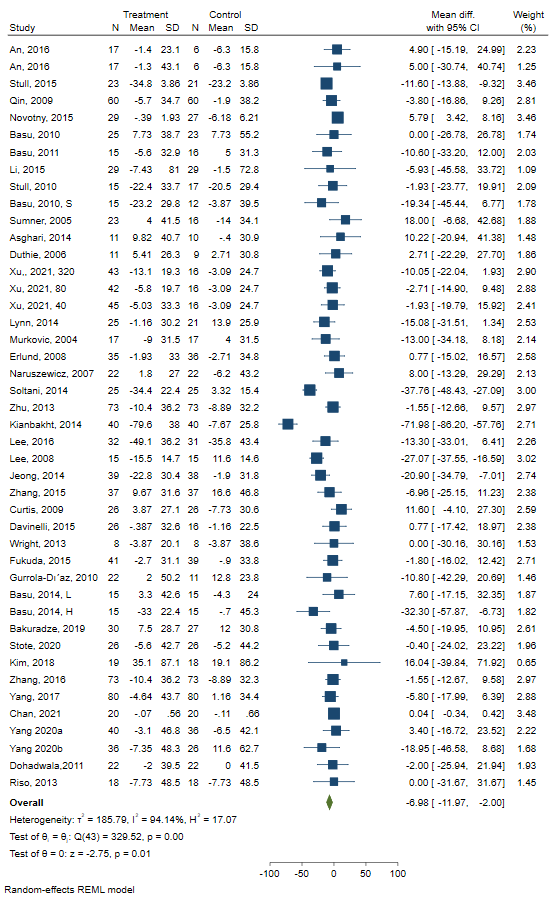


Figure 19S. Forest plot of the effect of anthocyanins compared with control on serum total cholesterol (mg/dL)


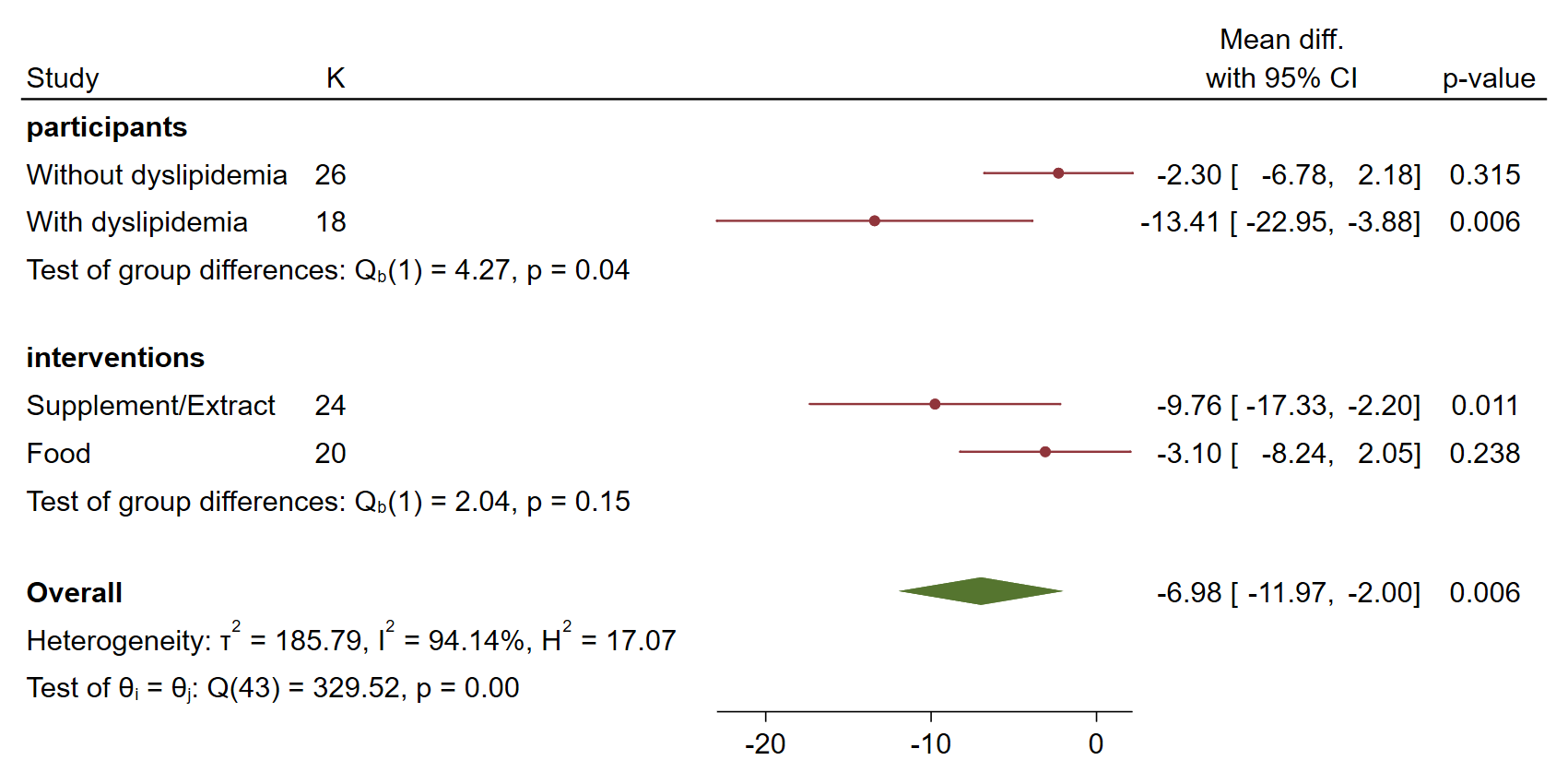


Figure 20S. Result of subgroup analysis of the effect of anthocyanins compared with control on serum total cholesterol (mg/dL)


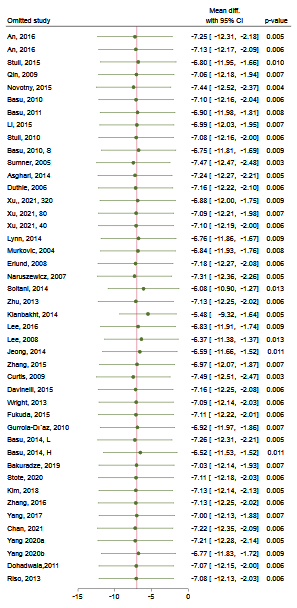


Figure 21S. The result of leave-one-out analysis of the effect of anthocyanin on serum total cholesterol concentrations (mg/dL)


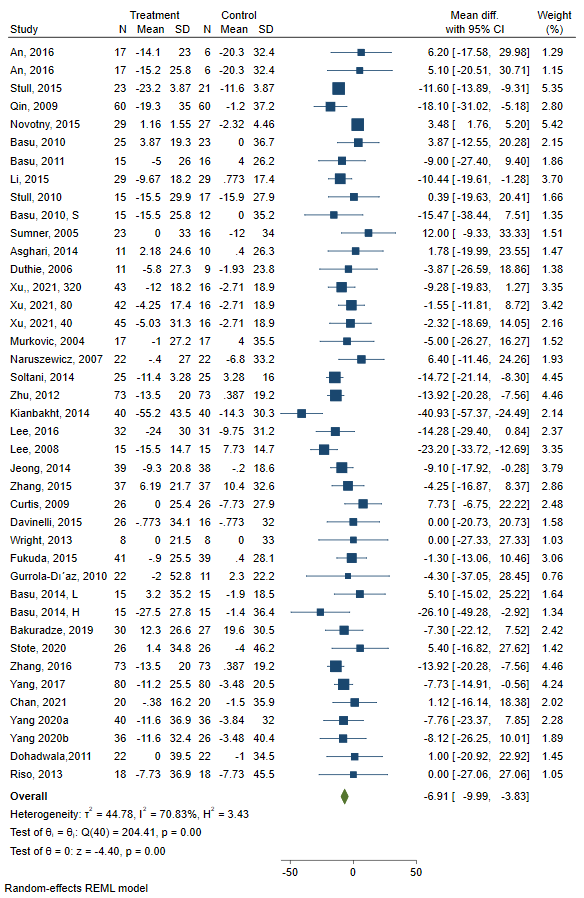


Figure 22S. Forest plot of the effect of anthocyanins compared with control on serum LDL-C concentrations (mg/dL)


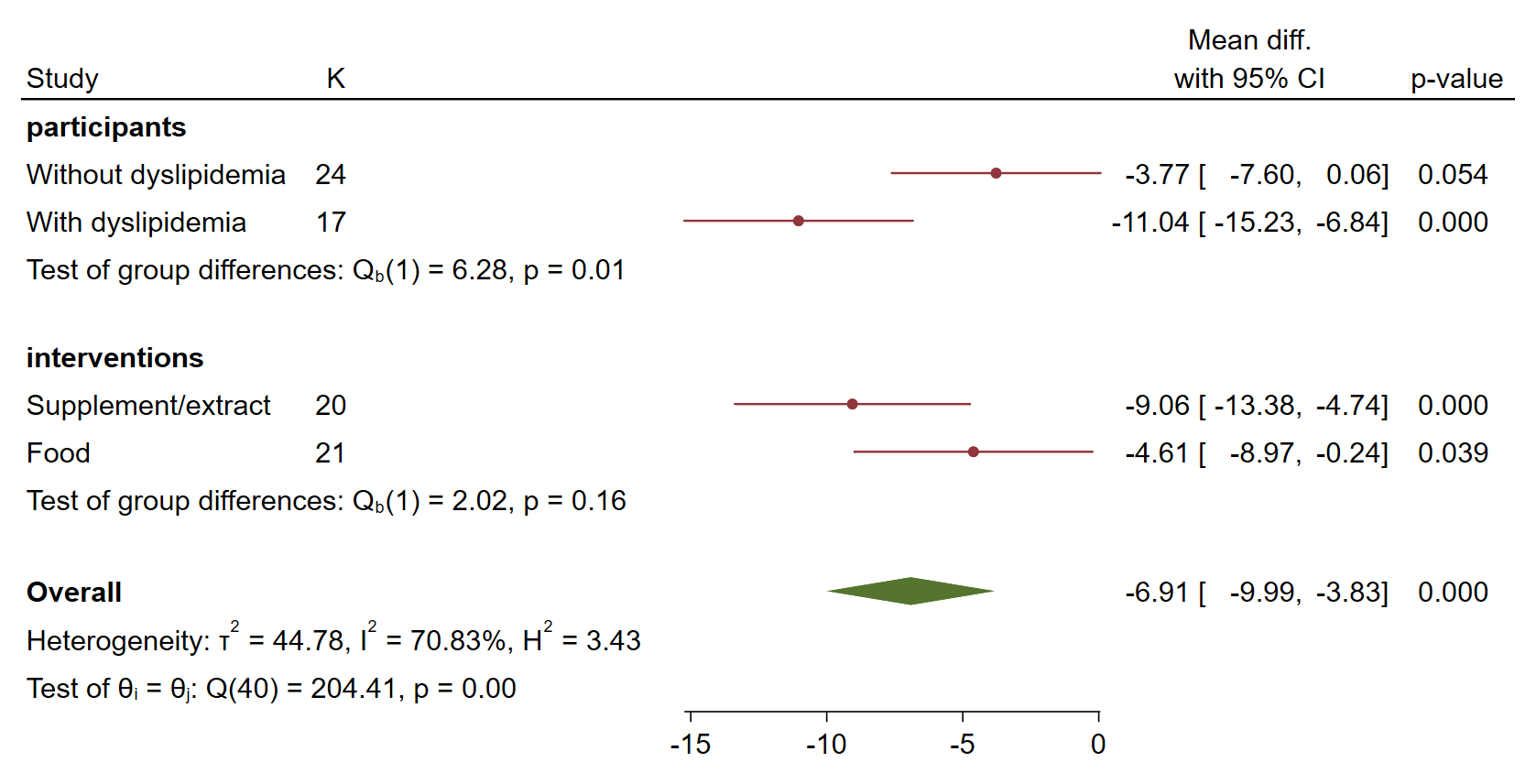


Figure 23S. The result of subgroup analysis of the effect of anthocyanins compared with control on serum LDL-C concentrations (mg/dL)


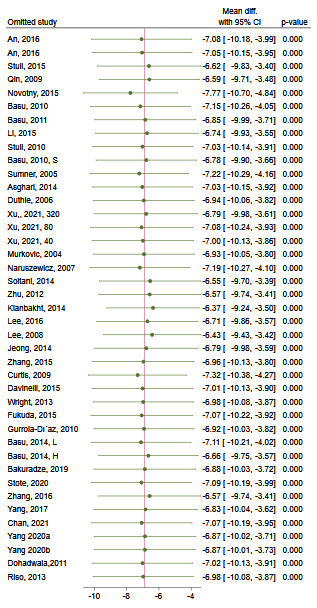


Figure 24S. The result of leave-one-out analysis of the effect of anthocyanin on serum LDL-C concentrations (mg/dL)


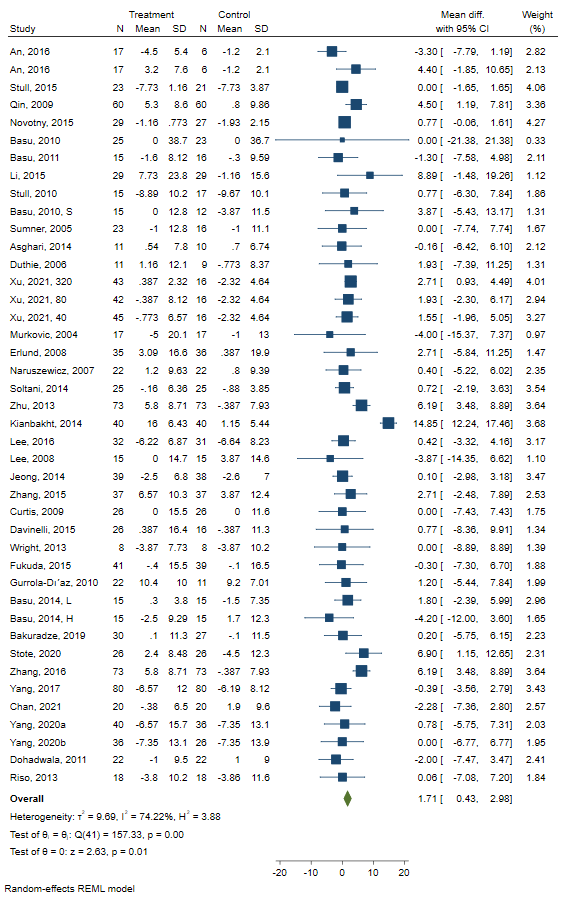


Figure 25S. Forest plot of the effect of anthocyanins compared with control on serum HDL-C concentrations (mg/dL)


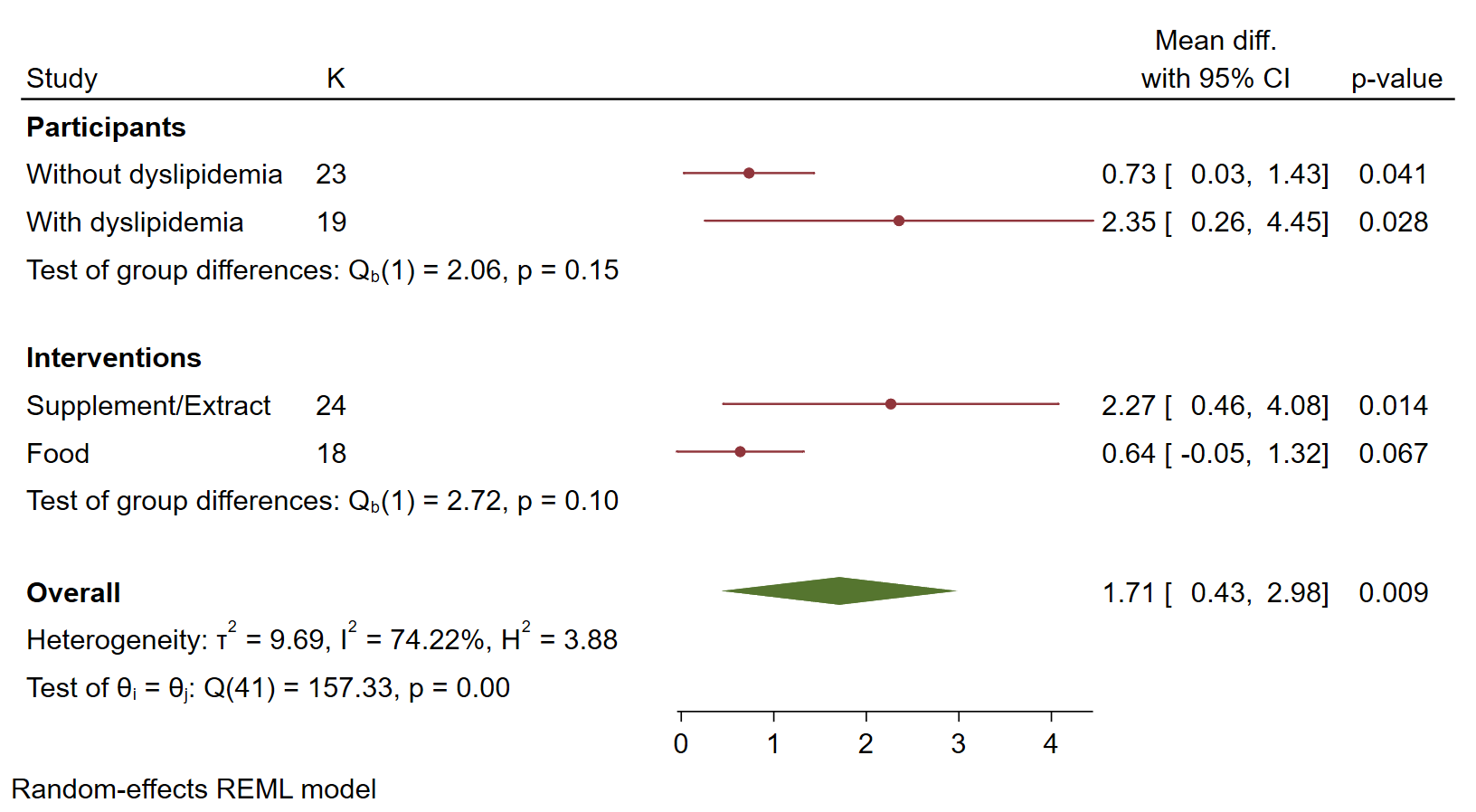


Figure 26S. Result of subgroup analysis of the effect of anthocyanins compared with control on serum HDL-C concentrations (mg/dL)


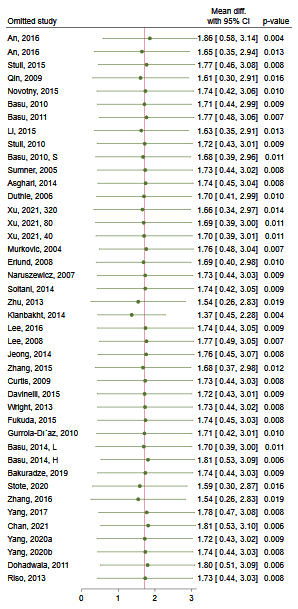


Figure 27S. The result of leave-one-out analysis of the effect of anthocyanin on serum HDL-C concentrations (mg/dL)


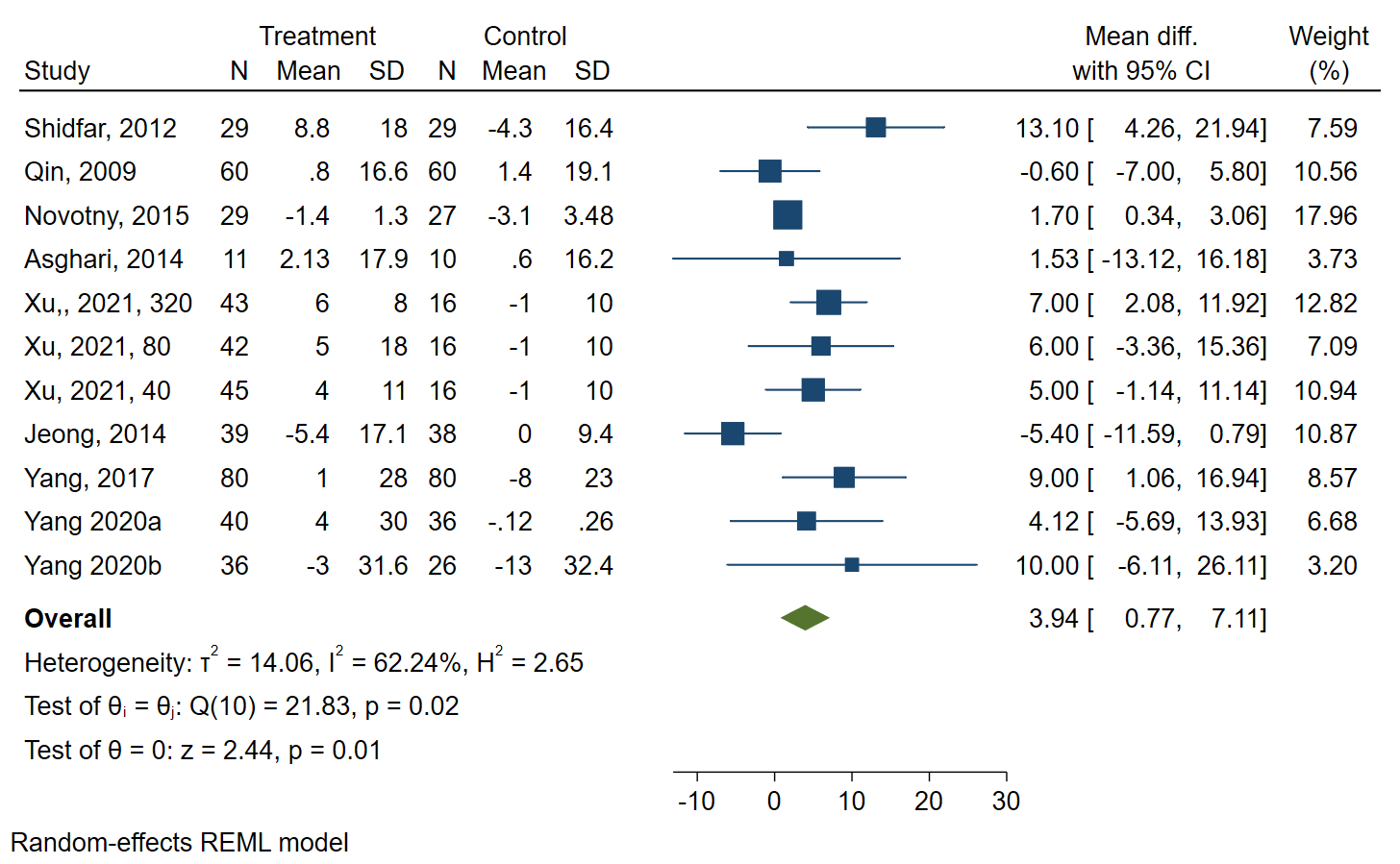


Figure 28S. Forest plot of the effect of anthocyanins compared with control on serum Apo A concentrations (mg/dL)


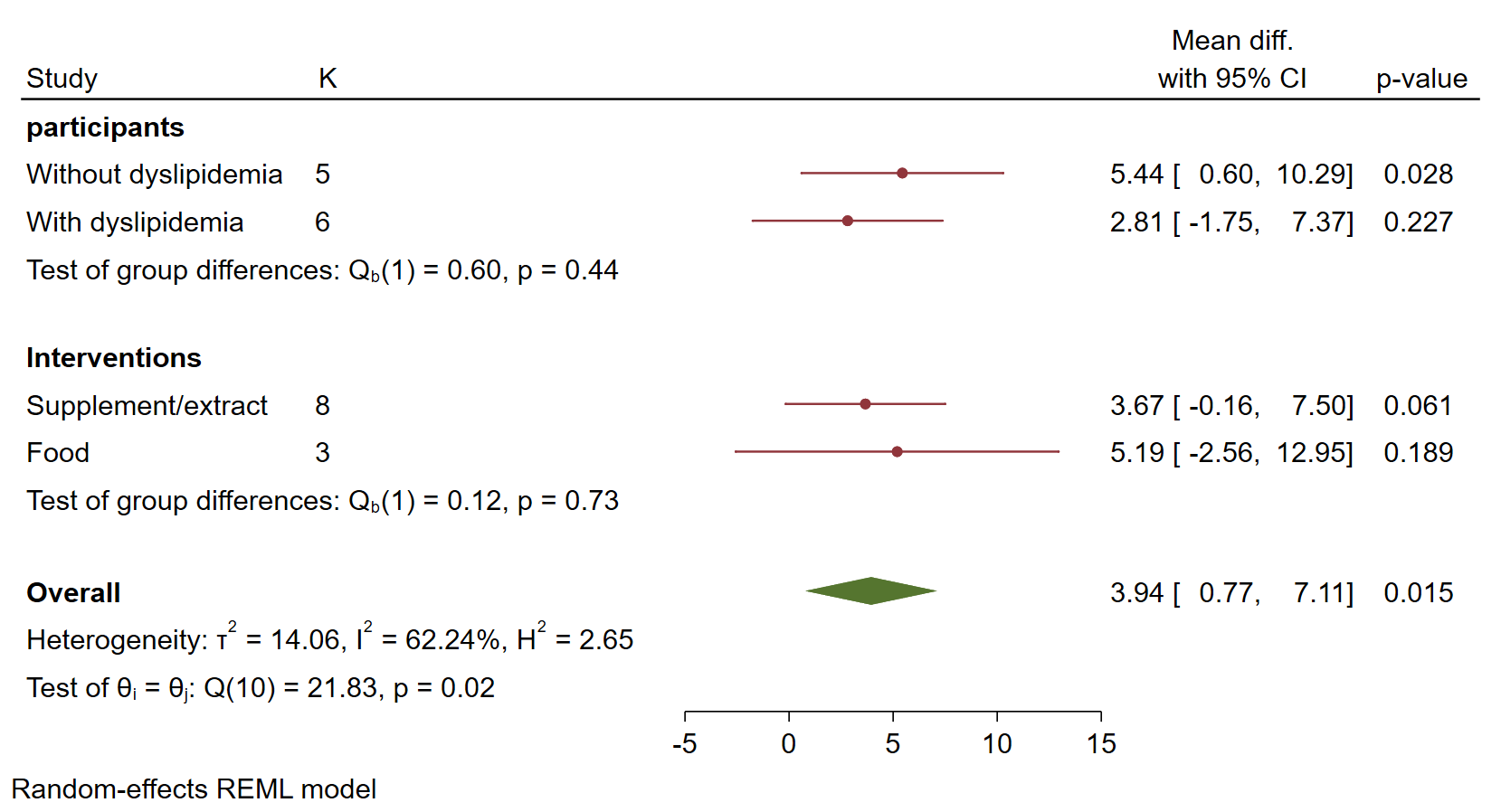


Figure 29S. Result of subgroup analysis of the effect of anthocyanins intake compared with control on serum Apo A concentrations (mg/dL)


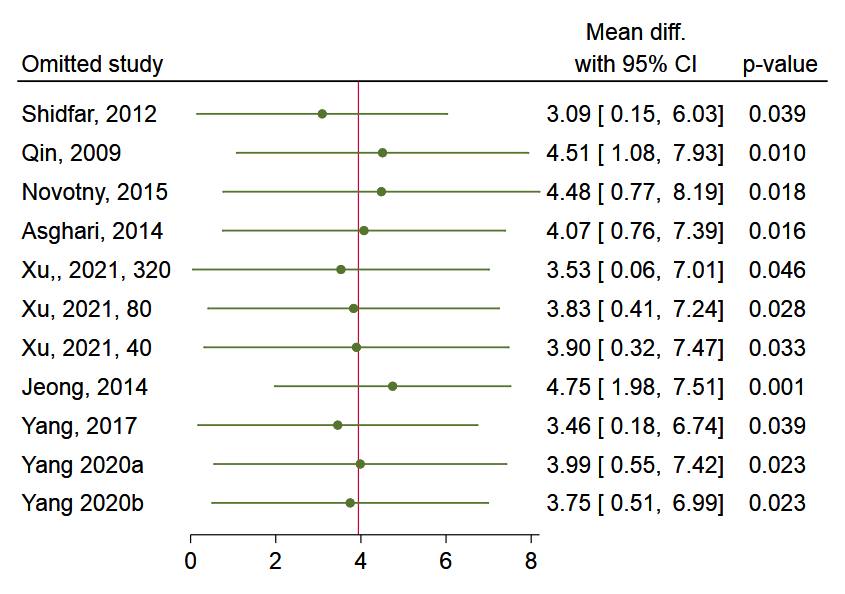


Figure 30S. The result of leave-one-out analysis of the effect of anthocyanin on serum Apo A concentrations (mg/dL)


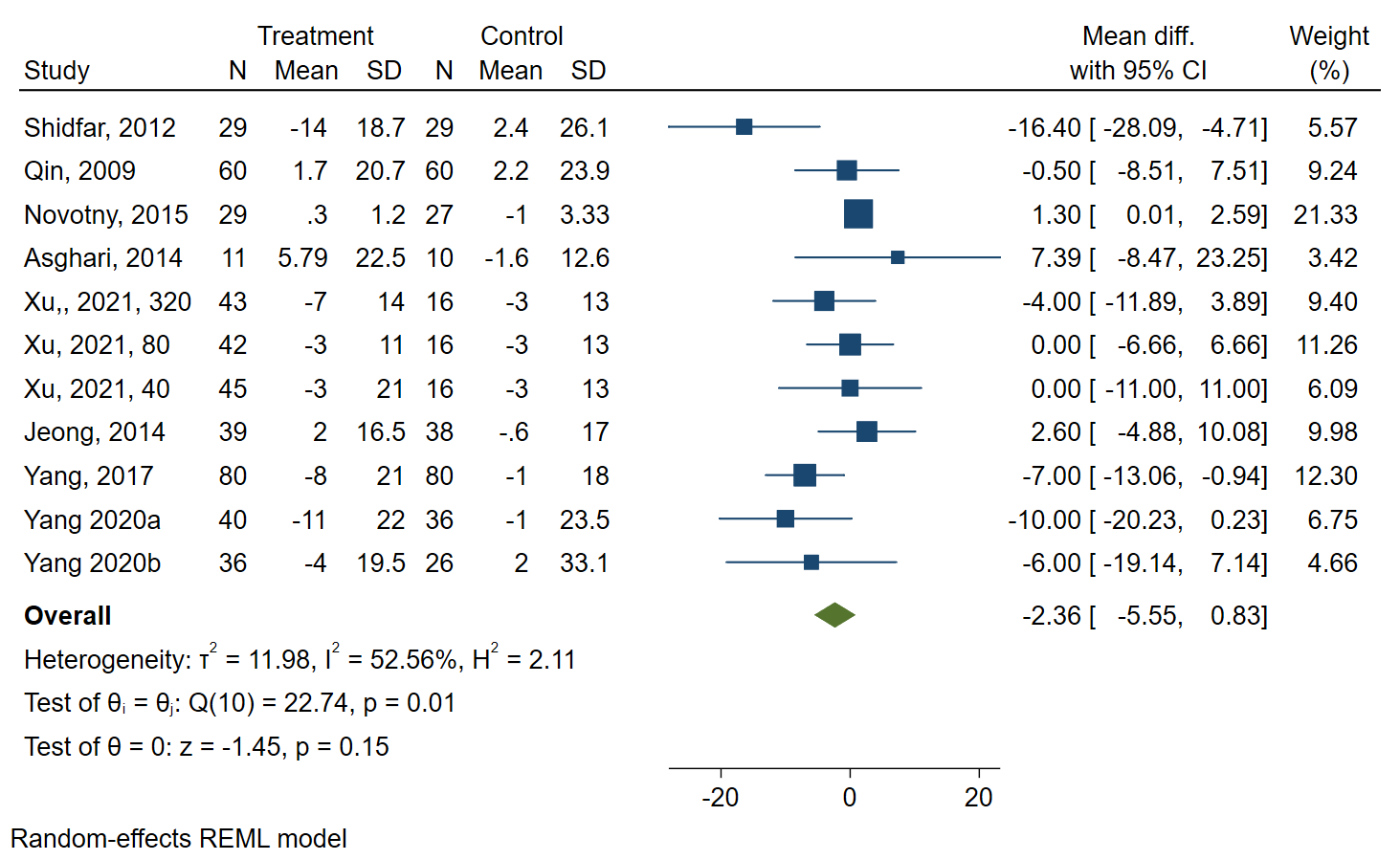


Figure 31S. Forest plot of the effect of anthocyanins compared with control on serum Apo B concentrations (mg/dL)


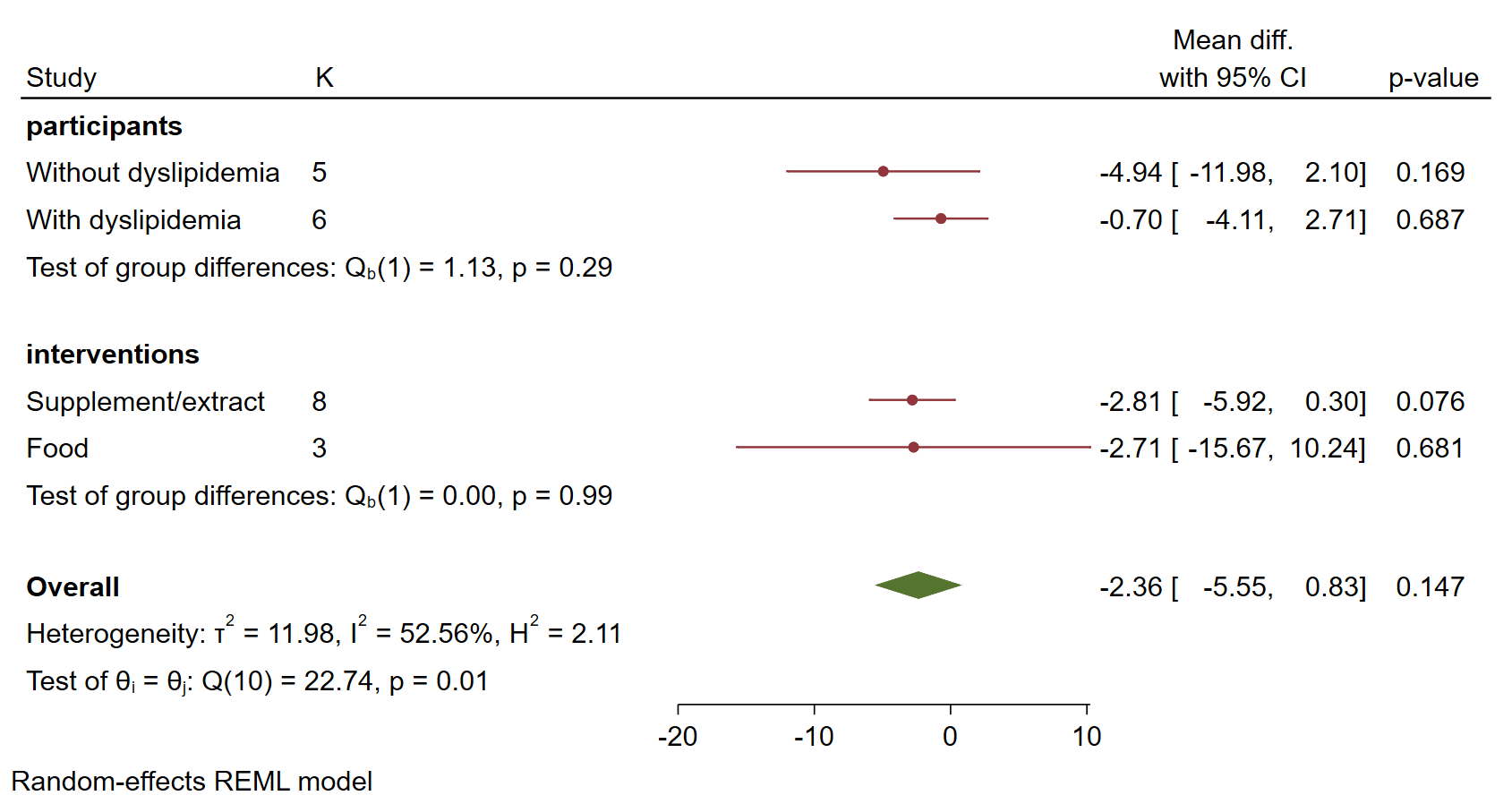


Figure 32S. Result of subgroup analysis of the effect of anthocyanins intake compared with control on serum Apo B concentrations (mg/dL)


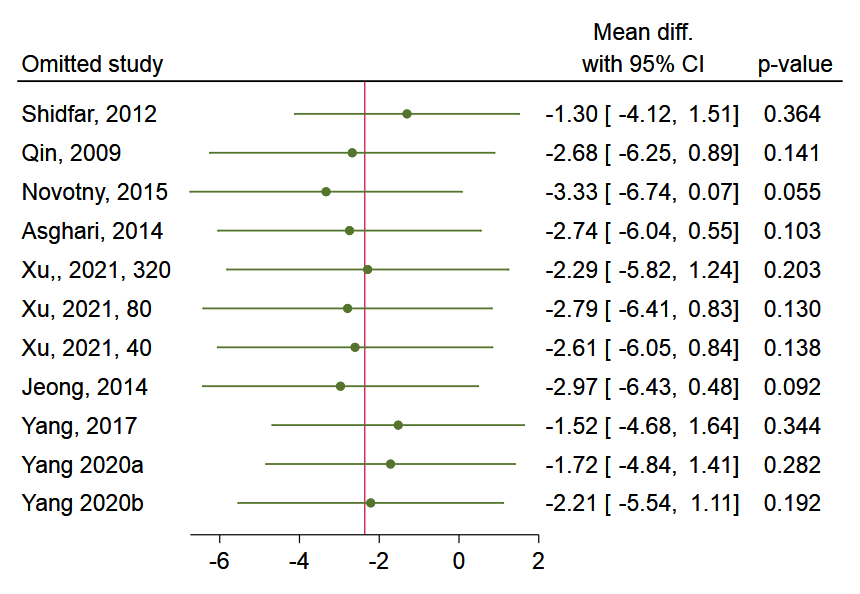


Figure 33S. The result of leave-one-out analysis of the effect of anthocyanin on serum Apo B concentrations (mg/dL)
